# Supplementary material for: Optimizing Synthetic miRNA Minigene Architecture for Efficient miRNA Hairpin Concatenation and Multi-target Gene Knockdown
Source: Mol Ther Nucleic Acids. 2018 Dec 14;14:351–63. doi: 10.1016/j.omtn.2018.12.004 (PMC6350225; doi:10.1016/j.omtn.2018.12.004)
Supplement: Document S1. Figures S1–S5 and Tables S1–S6 [file mmc1.pdf]

OMTN, Volume *14*

Supplemental Information

Optimizing Synthetic miRNA Minigene Architecture for Efficient miRNA Hairpin Concatenation and Multi-target Gene Knockdown

Francis Rousset, Patrick Salmon, Simon Bredl, Ophélie Cherpin, Marta Coelho, Renier Myburgh, Marco Alessandrini, Michael Perny, Marta Roccio, Roberto F. Speck, Pascal Senn, and Karl Heinz Krause

Supplementary Material

Supplementary Fig. 1. Impact of the length and biophysical features of the spacer on the knockdown efficiency. Plots showing the correlation between CCR5 knockdown and (a) the minimum free energy/length or (b) the GC content of the spacers described in this figure.

Supplementary Fig. 2. Overall strategy for the design of efficient miRGE concatenate targeting NOX3 activity. (a) Candidate siRNA sequences are design of in silico and selected on their ability to knock-down the target mRNA (NOX3, $p22^{phox}$ or NOXO1). The most active siRNA sequence is then cloned in the miRGE backbone to construct the SMIG. Validation of the SMIG efficiency is verified on the same target expressing cell line. (b) Potency of 8 siRNA candidates, targeting mRNA encoding for the NOX3 complex (NOX3, $p22^{phox}$ and NoxO1) were assessed with respect to NOX3 activity by the amplex red method. (c) Effect of the most efficient sequence (targeting $p22^{phox}$) on NOX activity was verified by Western Blot. (d-e) Efficiency of the triple hairpin concatenate SMIG designed with the most active siRNA sequence was checked on the NOX3 expressing cell line either by QPCR (d) or by measurement of the production of ROS (e).

Supplementary Figure 3. Calculation of the efficiency of concatenation of the miRGE hairpins. (a) Table showing the formula allowing the calculation of the efficiency of concatenation (E) for GFP, MGST2 and H2B spacers. For the calculation of the concatenation efficiency, CCR5 knockdown with a single miRGE hairpin (knock-down potency (KP)) and with triple miRGE concatenate (concatenation potency (CP)) were considered. (b) Table summarizing the knock-down potency (KP), the concatenation efficiency (E) and their product, the general vector efficiency. (c) The former one is also displayed on the histogram.

Supplementary Figure 4. Engraftement rate of human hematopoietic stem cells in NOD/SCID mice, 23 weeks following transplantation. FACS plot showing human circulating leukocytes expressing human CD45 marker in (a) a mouse transplanted with untrannsduced HSC or (b) the 6 mice transplanted with HSC transduced with the triple hairpin concatenate targeting CCR5. Engraftment rate is between 12.4% and 44%.

Supplementary Figure 5. Identification of human CD4+ T cells from the transplanted mice blood. Total lymphocytes were selected according to their FSC/SSC coordinates (a). Following doublet elimination (b), human leukocytes were selected with human CD45 staining (c), then human lymphocytes with human CD3 antibody (d). Finally, CD4+ T cells were discriminated from CD8+ T cells with human CD4 and human CD8 antibodies (e).

Supplementary table 1. Biophysical features of the spacer sequences used in the vector constructs and putative function of translated proteins in the recipient cells.

Supplementary table 2. Lentivectors used in the study.

Supplementary table 3. Table of qPCR primers.

Supplementary table 4. Table of cloning primers.

Supplementary table 5. Table of DNA template for miRGE cloning.

Supplementary table 6. Sequences of the spacers

Supplementary Fig. 1

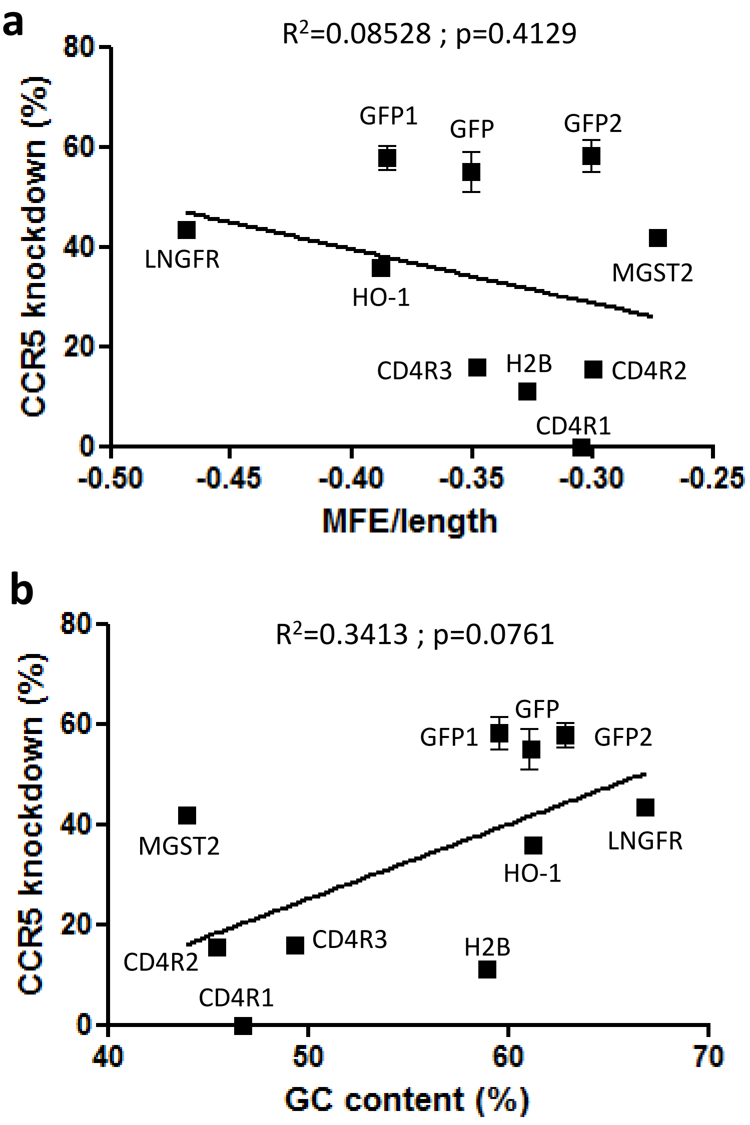

Supplementary Fig. 2

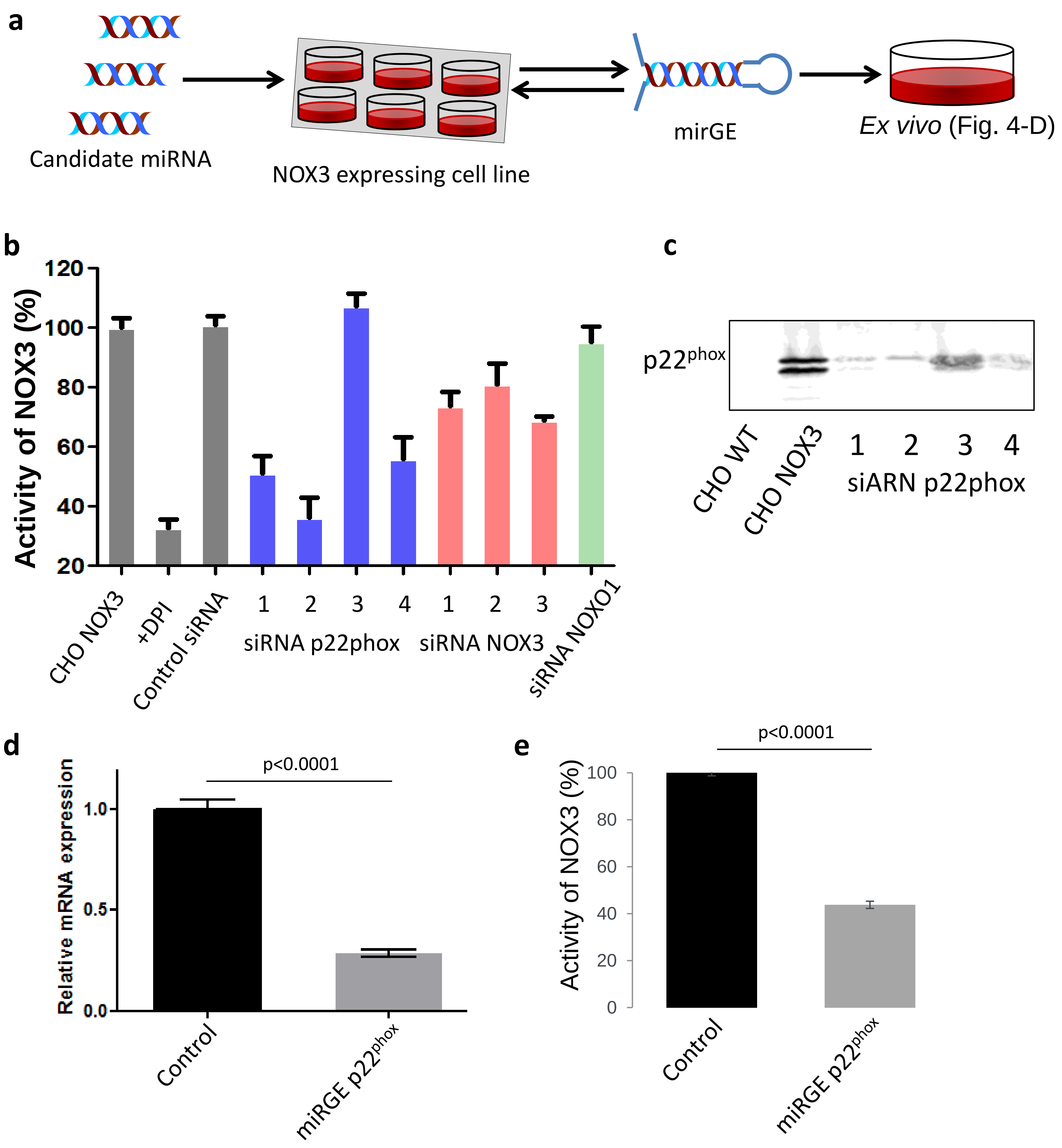

Supplementary Fig. 3

Concatenation potency = CP (%)	E= Concatenation efficiency with 3 hairpins
Knockdown potency = KP (%)	
$CP=(KP)^E$	If E=3 concatenation efficiency is 100%
$E=\ln(CP)/\ln(KP)$	If E=1 concatenation efficiency is 0%

Supplementary Fig. 4

a

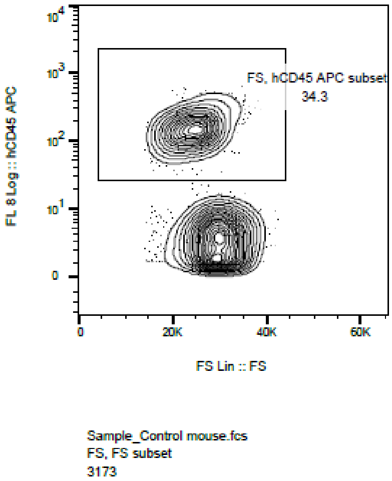

b

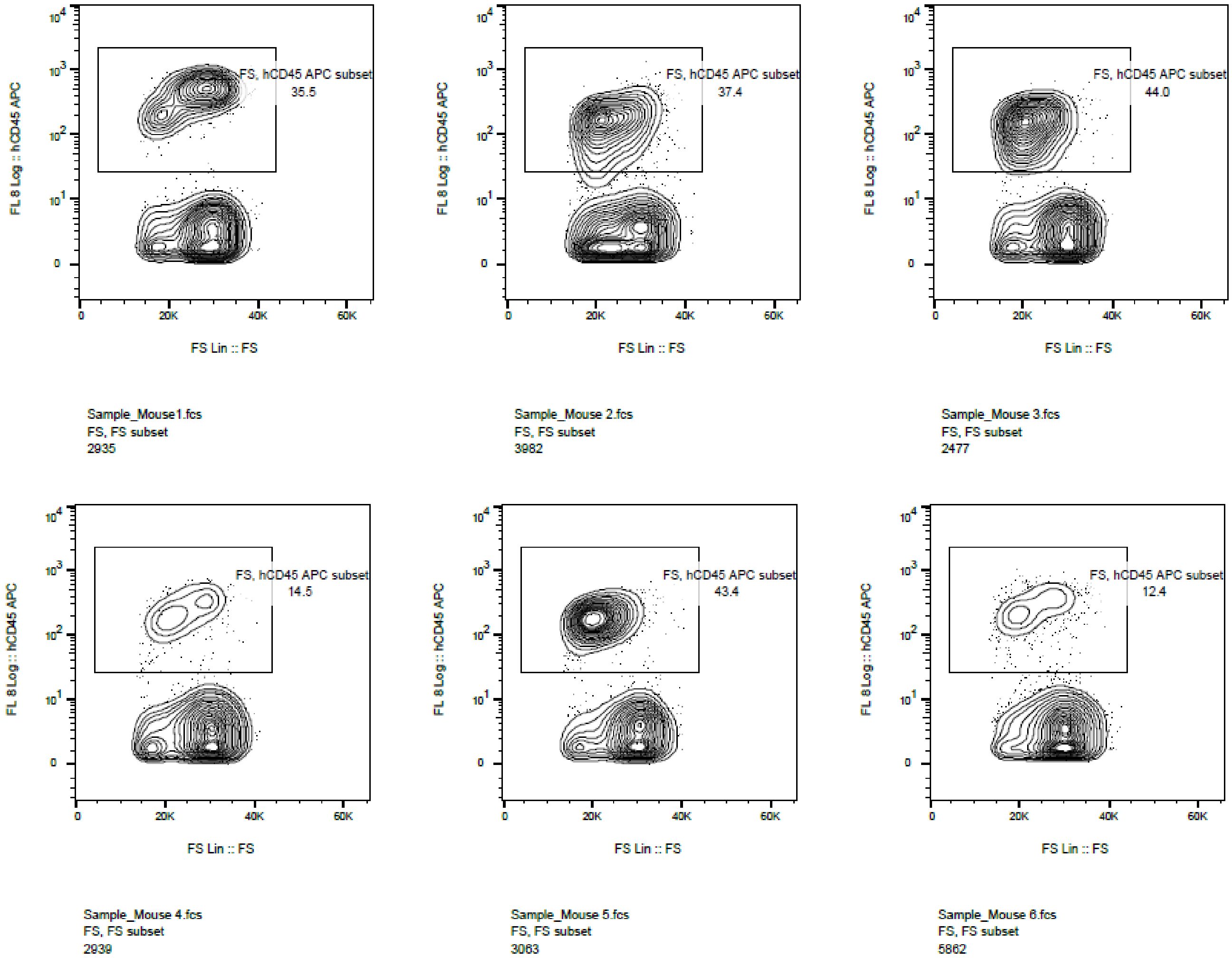

Supplementary Fig. 5

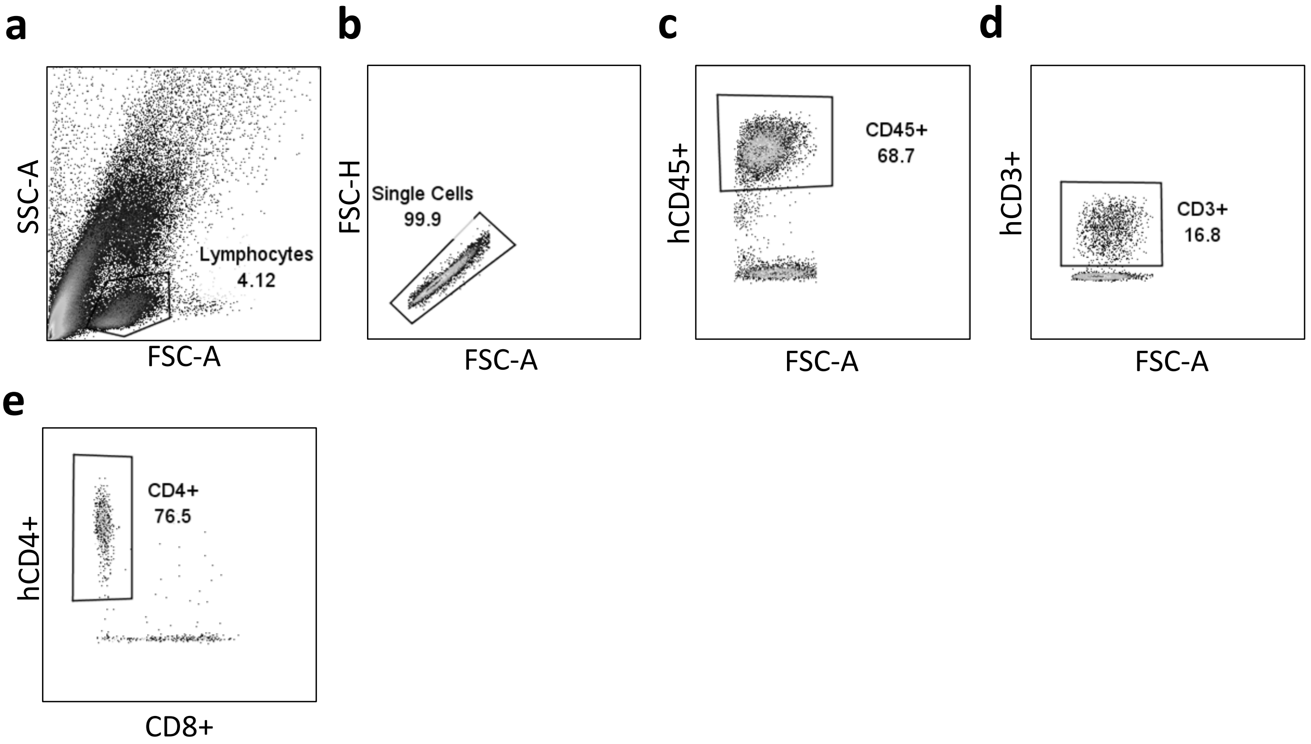

Supplementary table 1. Biophysical features of the spacer sequences

Spacer	Length (bp)	Predicted MFE Kcal/mol	% GC	coding	Comments
Green Fluorescent protein (GFP)<br>Truncated GFP1<br>GFP2	717<br>369<br>388	-264.6<br>-142.2<br>-116.4	61.11<br>62.87<br>59.53	YES	Reference spacer
Stop codons in the 3 reading frame after the start codon of GFP (stopGFP)	732	-	-	YES	non coding form
Microsomal Glutathion Transferase-2 (MGST2)	726	-197.7	43.92	YES	Resistance to Busulfan
Truncated form of the Nerve Growth Factor Receptor (deltaNGFR)	843	-397.7	66.86	YES	Pre-implantation selection
Heme Oxygenase (HO-1)	863	-336.2	61.24	YES	Cytoprotection
Histone2B (H2B)	378	-123.7	58.99	YES	Neutral
Amplicons from the 1st intron of the CD4 gene iCD41<br>iCD42<br>iCD43	793<br>620<br>380	-235.9<br>-189.2<br>-136	46.71<br>45.48<br>49.36	NO	Neutral ?

Supplementary table 2: Lentivectors used in this study

Lentivector	Titer (TU/mL)
pCWX-UBI-mcherry (control vector)	3.55E+06
pCWX-UBI-No spacer-mirGE CCR5-7-PGK-mCherry	1.02E+07
pCWX-UBI-GFP-mirGE CCR5-7-PGK-mCherry	4.66E+06
pCWX-UBI-mirGE CCR5-7-GFP-PGK-mCherry	1.17E+07
pCWX-UBI-MGST2-miRGE CCR5-7-PGK-mCherry	7.80E+05
pCWX-UBI-NGFR-miRGE CCR5-7-PGK-mCherry	1.94E+05
pCWX-UBI-CD4 R1-miRGE CCR5-7-PGK-mCherry	1.80E+06
pCWX-UBI-CD4 R2-miRGE CCR5-7-PGK-mCherry	2.76E+06
pCWX-UBI-CD4 R3-miRGE CCR5-7-PGK-mCherry	2.52E+06
pCWX-UBI-HO-1-miRGE CCR5-7-PGK-mCherry	1.60E+06
pCWX-UBI-H2B-miRGE CCR5-7-PGK-mCherry	1.22E+07
pCWX-UBI-stopGFP-miRGE CCR5-7-PGK-mCherry	3.54E+06
pCWX-UBI-GFPpart1-miRGE CCR5-7-PGK-mCherry	1.56E+06
pCWX-UBI-GFPpart2-miRGE CCR5-7-PGK-mCherry	1.59E+06
pCWX-UBI-MGST2-miRGE CYBA 222-PGK-mCherry	1.57E+06
pCWX-UBI-stopGFP-mirGE CYBA 222-PGK-mCherry	2.31E+06
pCWX-UBI- MGST2-mirGE-CCR5-777-PGK-mCherry	1.34E+07
pCWX-UBI- MGST2-mirGE-CCR5-777GFP-PGK-mCherry	1.20E+07
pCWX-UBI- MGST2-mirGE-CCR5-7777-PGK-mCherry	1.10E+05
pCWX-UBI- MGST2-mirGE-CCR5-7777GFP-PGK-mCherry	1.28E+07
pCWX-UBI-No spacer-mirGE CCR5-777-PGK-mCherry	6.81E+06
pCWX-UBI-mirGE CCR5-777-GFP-PGK-mCherry	1.10E+07
pCWX-UBI-H2B-miRGE CCR5-777-PGK-mCherry	5.06E+06
pCWX-UBI- stopGFP-mirGE-CCR5-777-PGK-mCherry	1.00E+06
pCWX-UBI- stopGFP-mirGE-CCR5-777GFP-PGK-mCherry	1.84E+05
pCWX-UBI- stopGFP-mirGE-CCR5-7777-PGK-mCherry	1.98E+06
pCWX-UBI- stopGFP-mirGE-CCR5-7777GFP-PGK-mCherry	1.12E+06
pCWX-UBI-stopGFP-miRGEGFP-PGK-mCherry	9.02E+05
pCWX-EFs-No spacer-mirGE CCR5-7-PGK-mCherry	9.25E+06
pCWX-EFs-GFP-mirGE CCR5-7-PGK-mCherry	1.21E+07
pCWX-EFs-GFP-mirGE CCR5-777-PGK-mCherry	1.27E+07
pCWX-EFs-mirGE CCR5-7-GFP-PGK-mCherry	6.82E+06
pCWX-EFs-mirGE CCR5-777-GFP-PGK-mCherry	4.75E+06
pCWX-EFs-No spacer-mirGE CCR5-777-PGK-mCherry	6.02E+06

Supplementary table 3: Table of qPCR primers used in this study

Amplicon	Primer forward 5’-3’	Primer reverse 5’-3’
Cyba (p22^{phox})	TGG ACG TTT CAC ACA GTG GT	TGG ACC CCT TTT TCC TCT TT
miRGE pri-miRNA	GGT GAT AGC AAT GTC AGC AGT GCC T	GTA GAG TAT GGT CAA CCT TAC TT
mature miRGE	LNA modified proprietary sequence (exiqon)	
GAG	GGA GCT AGA ACG ATT CGC AGT TA	GGT TGT AGC TGT CCC AGT ATT TGT C
EEF1a	TCC ACT TGG TCG CTT TGC T	CTT CTT GTC CAC AGC TTT GAT GA
GAPDH	TCC ATG ACA ACT TTG GCA TTG	CAG TCT TCT GGG TGG CAG TGA

Supplementary table 4: Table of cloning primers used in this study

Amplicon	Primer forward 5'-3' (AttB1-RcoRI)	Primer reverse 5'-3' (AttB2-XhoI)
GFP	GGG GAC AAG TTT GTA CAA AAA AGC AGG CTG AAT TCT GAG CAA GGG CGA GGA GCT GT	GGG GAC CAC TTT GTA CAA GAA AGC TGG GTC TCG AGC TTG TAC AGC TCG TCC ATG CCG
stopGFP	GGG GAC AAG TTT GTA CAA AAA AGC AGG CTT CTA GAA TGG ATG TAA GTA GGT GAG TGA GCA	GGG GAC CAC TTT GTA CAA GAA AGC TGG GTC TCG AGC TTG TAC AGC TCG TCC ATG CCG AGA
GFPpart1	GGG GAC AAG TTT GTA CAA AAA AGC AGG CTG AAT TCT GAG CAA GGG CGA GGA GCT GT	GGG GGC TCG AGT CGC CCT CGA ACT TCA CCT CG
GFPpart2	GGG GGG AAT TCC ACC CTG GTG AAC CGC ATC GA	GGG GAC CAC TTT GTA CAA GAA AGC TGG GTC TCG AGC TTG TAC AGC TCG TCC ATG CCG
lNGFR	GGG GAC CAC TTT GTA CAA GAA AGC TGG GTC TCG AGC TAG AGG ATC CCC CTG TTC CAC CT	GGG GAC AAG TTT GTA CAA AAA AGC AGG CTG AAT TCT CAC CAT GGG GGC AGG TGC CAC CGG
HO-1	GGG GAC AAG TTT GTA CAA AAA AGC AGG CTG AAT TCT CAC CAT GGA GCG TCC GCA ACC CGA	GGG GAC CAC TTT GTA CAA GAA AGC TGG GTC TAC AGC AAC TGT CGC CAC C
CD4R1	GGG GAC AAG TTT GTA CAA AAA AGC AGG CTG AAT TCT AAT AGT GAC CAC TCC TGG CTA ATT TTT GTA TTT TCA GTA GAG ATA GGG	GGG GAC CAC TTT GTA CAA GAA AGC TGG GTC TCG AGG GTG AAA CCC TTC TCT ACT AAA AAT ACA AAA TTA GCC GGG CAC A
CD4R2	-	GGG GAC CAC TTT GTA CAA GAA AGC TGG GTC TCG AGC CGC ACT CCA GCC TCG GCG ACA GAG CAA GAC TCT ATC TCA
CD4R3	-	GGG GAC CAC TTT GTA CAA GAA AGC TGG GTC TCG AGT CGG GAG TAC GAG ACC AGC CTG GCC AAC ATA GTG AAA TCC
H2B	GGG GAC AAG TTT GTA CAA AAA AGC AGG CTG AAT TCA TGC CAG AGC CAG CGA AGT C	GGG GAC CAC TTT GTA CAA GAA AGC TGG GTC TCG AGG TGT ACT TGG TGA CGG CCT TA
miRGE 1st hairpin	CAG AAG GGG ATC CAT CGA TAC TAG TGG TGA TAG CAA TGT CAG CAG TGC CT	AGT AGC TTC TAG AGT AGA GTA TGG TCA ACC TTA CTT
miRGE 2nd hairpin	CAG AAG GGG ATC CGG TGA TAG CAA TGT CAG CAG TGC CT	AGT AGC TAC TAG TGT AGA GTA TGG TCA ACC TTA CTT
miRGE 3rd hairpin	CAG AAG GCT CGA GGG TGA TAG CAA TGT CAG CAG TGC CT	AGT AGC TGG ATC CGT AGA GTA TGG TCA ACC TTA CTT

Supplementary table 5: Table of DNA template for miRGE cloning

	Template 5'-3'
miRGE CCR5-7	GGT GAT AGC AAT GTC AGC AGT GCC TTC ATA GAT TGG ACT TGA CAC TTG TGA AGC CAC AGA TGA AGT GTC AAG CCC AAT CTA TGC AAG TAA GGT TGA CCA TAC TCT AC
miRGE GFP	GGT GAT AGC AAT GTC AGC AGT GCC TAG TTC ACC TTG ATG CCG TTC TTG TGA AGC CAC AGA TGA AGA ACG GCA CCA AGG TGA ACC AAG TAA GGT TGA CCA TAC TCT AC
miRGE p22phox	GGT GAT AGC AAT GTC AGC AGT GCC TAC ATG GCC CAC TCG ATC TGC CCG TGA AGC CAC AGA TGG GGC AGA TCG CGT GGG CCA TGC AAG TAA GGT TGA CCA TAC TCT AC

Supplementary table 6: sequences of the spacers

stopGFP

aattcatggatgtaagtaggtgagtgagcaagggcgaggagctgttcaccggggtggtgcccatcctggtcgagctggacggcgacgtaaacggccacaagttcagcgtgtccggcgagggcgag ggcgatgccac
ctacggcaagctgaccctgaagttcatctgcaccaccggcaagctgcccgtgccctggcccaccctcgtgaccaccctgacctacggcgtgcagtgcttcagccgctaccccgaccacatgaagc agcacgacttc
ttcaagtccgccatgcccgaaggctacgtccaggagcgcaccatcttcttcaaggacgacggcaactacaagacccgcgccgaggtgaagttcgagggcgacaccctggtgaaccgcatcgagct gaagggcatcg
acttcaaggaggacggcaacatcctggggcacaagctggagtacaactacaacagccacaacgtctatatcatggccgacaagcagaagaacggcatcaaggtgaacttcaagatccgccacaac atcgaggacgg
cagcgtgcagctcgccgaccactaccagcagaacacccccatcggcgacggccccgtgctgctgcccgacaaccactacctgagcacccagtccgccctgagcaaagaccccaacgagaagcgcg atcacatggtc
ctgctggagttcgtgaccgccgccgggatcactctcggcatggacgagctgtacaagtaaagcggcctgaatcgccagtgtc

GFP1

aattcatggatgtaagtaggtgagtgagcaagggcgaggagctgttcaccggggtggtgcccatcctggtcgagctggacggcgacgtaaacggccacaagttcagcgtgtccggcgagggcgag ggcgatgccac
ctacggcaagctgaccctgaagttcatctgcaccaccggcaagctgcccgtgccctggcccaccctcgtgaccaccctgacctacggcgtgcagtgcttcagccgctaccccgaccacatgaagc agcacgacttc
ttcaagtccgccatgcccgaaggctacgtccaggagcgcaccatcttcttcaaggacgacggcaactacaagacccgcgccgaggtgaagttcgagggcgacc

GFP2

Aattccaccctggtgaaccgcatcgagctgaagggcatcgacttcaaggaggacggcaacatcctggggcacaagctggagtacaactacaacagccacaacgtctatatcatggccgacaagca gaagaacggca
tcaaggtgaacttcaagatccgccacaacatcgaggacggcagcgtgcagctcgccgaccactaccagcagaacacccccatcggcgacggccccgtgctgctgcccgacaaccactacctgagc acccagtccgc
cctgagcaaagaccccaacgagaagcgcgatcacatggtcctgctggagttcgtgaccgccgccgggatcactctcggcatggacgagctgtacaagtaaagcggcctgaatcgccagtgtc

H2B

Atgccagagccagcgaagtctgctcccgccccgaaaaagggctccaagaaggcggtgactaaggcgcagaagaaaggcggcaagaagcgcaagcgcagccgcaaggagagctattccatctatgt gtacaaggttc
tgaagcaggtccaccctgacaccggcatttcgtccaaggccatgggcatcatgaattcgtttgtgaacgacattttcgagcgcatcgcaggtgaggcttcccgcctggcgcattacaacaagcgc tcgaccatcac
ctccagggagatccagacggccgtgcgcctgctgctgcctggggagttggccaagcacgccgtgtccgagggtactaaggccgtcaccaagtacaccagcgctaag

NGFR

Aattctcaccatgggggcaggtgccaccggccgcgccatggacgggccgcgcctgctgctgttgctgcttctgggggtgtcccttggaggtgccaaggaggcatgccccacaggcctgtacacac acagcggtgag
tgctgcaaagcctgcaacctgggcgagggtgtggcccagccttgtggagccaaccagaccgtgtgtgagccctgcctggacagcgtgacgttctccgacgtggtgagcgcgaccgagccgtgcaa gccgtgcaccg
agtgcgtggggctccagagcatgtcggcgccgtgcgtggaggccgacgacgccgtgtgccgctgcgcctacggctactaccaggatgagacgactgggcgctgcgaggcgtgccgcgtgtgcgag gcgggctcggg
cctcgtgttctcctgccaggacaagcagaacaccgtgtgcgaggagtgccccgacggcacgtattccgacgaggccaaccacgtggacccgtgcctgccctgcaccgtgtgcgaggacaccgagc gccagctccgc
gagtgcacacgctgggccgacgccgagtgcgaggagatccctggccgttggattacacggtccacacccccagagggctcggacagcacagcccccagcacccaggagcctgaggcacctccaga acaagacctca
tagccagcacggtggcaggtgtggtgaccacagtgatgggcagctcccagcccgtggtgacccgaggcaccaccgacaacctcatccctgtctattgctccatcctggctgctgtggttgtgggc cttgtggccta
catagccttcaagaggtggaacaggggggatcctctagc

MGST2

aattcggcacgagggacttctgttccagagcaaaggtcattcagccgcttgaatcagccttttcccccacccggtccccaactttgtttacccgataaggaaggtcagcattcaaagtcaagaa gcgccatttat
cttcccgtgcgctctacaaatagttccgtgagaaagatggccgggaactcgatcctgctggctgctgtctctattctctcggcctgtcagcaaagttatttgctttgcaagttggaaaggcaag attaaaataca
aagttacgcccccagcagtcactgggtcaccagagtttgagagagtatttcgggcacaacaaaactgtgtggagttttatcctatattcataattacattgtggatggctgggtggtatttcaac caagtttttgc
tacttgtctgggtctggtgtacatatatggccgtcacctatacttctggggatattcagaagctgctaaaaaacggatcaccggtttccgactgagtctggggattttggccttgttgaccctcc taggtgccctg
ggaattgcaaacagctttctggatgaatatctggacctcaatattgccaagaaactgaggcggcaattctaacttttctcttccctttaatgcttgcagaagctgttcccaccatgaaggtaat atggtatcatt
tgttaaataaaaataaagtctttattctgttaaaaaaaaaaaaaaaaaac

HO-1

Aattcatggagcgtccgcaacccgacagcatgccccaggatttgtcagaggccctgaaggaggccaccaaggaggtgcacacccaggcagagaatgctgagttcatgaggaactttcagaagggc caggtgacccg
agacggcttcaagctggtgatggcctccctgtaccacatctatgtggccctggaggaggagattgagcgcaacaaggagagcccagtcttcgcccctgtctacttcccagaagagctgcaccgca aggctgccctg
gagcaggacctggccttctggtacgggccccgctggcaggaggtcatcccctacacaccagccatgcagcgctatgtgaagcggctccacgaggtggggcgcacagagcccgagctgctggtggc ccacgcctaca
cccgctacctgggtgacctgtctgggggccaggtgctcaaaaagattgcccagaaagccctggacctgcccagctctggcgagggcctggccttcttcaccttccccaacattgccagtgccacc aagttcaagca
gctctaccgctcccgcatgaactccctggagatgactcccgcagtcaggcagagggtgatagaagaggccaagactgcgttcctgctcaacatccagctctttgaggagttgcaggagctgctga cccatgacacc
aaggaccagagcccctcacgggcaccagggcttcgccagcgggccagcaacaaagtgcaagattctgcccccgtggagactcccagagggaagcccccactcaacacccgctcccaggctccgct tctccgatggg
tccttacactcagctttctggtggcgacagttgctgtagggctttatgccatgtgac

iCD41

aattctaatagtgaccactcctggctaatttttgtattttcagtagagatagggtttcactatgttggccaggctggtctccaactcctgacctaaagtgatccacccaccttggtttcccaaag tgctgggatta
caggcgtgagccaccgtgcctggacatatatctatctttttttttttttgagatggagtctcgctctgttgcccaggctggagtgcagtggcgtgatttcggctcactgcaacctccgcctcccgg gttcaagtgat
tctcctgcctcagcctcccaagtagctgagattacagacgtgcgtcaccatgcccagctaatttttgtatttttagtagagatgggatttcactatgttggccaggctggtctcgtactcccgac ctcaggtgatc
cacttgccttggcctcccaaagtgctggaattacaggtgtgagccactgcatccggccttatatatctatcttgtctgtctgactgtctaatctaattcatctattttatctgtttatcttatct atcatctattt
atctaatctatctgtctgtatgtctgttttttttttgttttttttttttttttgagatagagtcttgctctgtcgccgaggctggagtgcggtggcgcgatctcagctcactgctgaacctccgc ctcctgggttc
taagcgattctcctgcctcaatctttggagtagctgggattacaggcccgtaccactgtgcccggctaattttgtatttttagtagagaagggtttcaccc

iCD42

aattctaatagtgaccactcctggctaatttttgtattttcagtagagatagggtttcactatgttggccaggctggtctccaactcctgacctaaagtgatccacccaccttggtttcccaaagtgctgggatta
caggcgtgagccaccgtgcctggacatatatctatctttttttttttttgagatggagtctcgctctgttgcccaggctggagtgcagtggcgtgatttcggctcactgcaacctccgcctcccgg gttcaagtgat
tctcctgcctcagcctcccaagtagctgagattacagacgtgcgtcaccatgcccagctaatttttgtatttttagtagagatgggatttcactatgttggccaggctggtctcgtactcccgac ctcaggtgatc
cacttgccttggcctcccaaagtgctggaattacaggtgtgagccactgcatccggccttatatatctatcttgtctgtctgactgtctaatctaattcatctattttatctgtttatcttatct atcatctattt
atctaatctatctgtctgtatgtctgttttttttttgttttttttttttttttgagatagagtcttgctctgtcgccgaggctggagtgcggc

iCD43

aattctaatagtgaccactcctggctaatttttgtattttcagtagagatagggtttcactatgttggccaggctggtctccaactcctgacctaaagtgatccacccaccttggtttcccaaag tgctgggatta
caggcgtgagccaccgtgcctggacatatatctatctttttttttttttgagatggagtctcgctctgttgcccaggctggagtgcagtggcgtgatttcggctcactgcaacctccgcctcccgg gttcaagtgat
tctcctgcctcagcctcccaagtagctgagattacagacgtgcgtcaccatgcccagctaatttttgtatttttagtagagatgggatttcactatgttggccaggctggtctcgtactcccgac
