# Supplementary material for: Optimizing Synthetic miRNA Minigene Architecture for Efficient miRNA Hairpin Concatenation and Multi-target Gene Knockdown
Source: Mol Ther Nucleic Acids. 2018 Dec 14;14:351–63. doi: 10.1016/j.omtn.2018.12.004 (PMC6350225; doi:10.1016/j.omtn.2018.12.004)
Supplement: Document S2. Article plus Supplemental Information [file mmc2.pdf]

Molecular Therapy
Nucleic Acids
Original Article

Optimizing Synthetic miRNA Minigene Architecture for Efficient miRNA Hairpin Concatenation and Multi-target Gene Knockdown

Francis Rousset,[1] Patrick Salmon,[2] Simon Bredl,[3] Ophélie Cherpin,[1] Marta Coelho,[2] Renier Myburgh,[4] Marco Alessandrini,[1] Michael Perny,[5] Marta Roccio,[5] Roberto F. Speck,[3] Pascal Senn,[1,6] and Karl Heinz Krause[1]

[1]Department of Pathology and Immunology, Faculty of Medicine, University of Geneva, Geneva, Switzerland; [2]Department of Neurosciences, Faculty of Medicine, University of Geneva, Geneva, Switzerland; [3]Department of Infectious Diseases and Hospital Epidemiology, University Hospital of Zurich, Zurich, Switzerland; [4]Division of Hematology, University Hospital of Zurich, Zurich, Switzerland; [5]Department of Otorhinolaryngology, Inselspital and University of Bern, Bern, Switzerland; [6]Department of Head and Neck Surgery, University Hospital of Geneva, Geneva, Switzerland

Synthetic microRNA (miRNA) minigenes (SMIGs) have a major potential for molecular therapy; however, their optimal architecture still needs to be determined. We have previously optimized the stem structure of miRNA hairpins for efficient gene knockdown. Here, we investigate the overall architecture of SMIGs driven by polymerase II-dependent promoters. When miRNA hairpins were placed directly behind the promoter, gene knockdown was inefficient as compared with constructs containing an intercalated sequence ("spacer"). Spacer sequence was relevant for knockdown efficiency and concatenation potential: GFP-based sequences (even when truncated or including stop codons) were particularly efficient. In contrast, a spacer of similar length based on a CD4 intronic sequence was entirely inefficient. Spacer sequences influenced miRNA steady-state levels without affecting transcript stability. We demonstrate that with an optimized spacer, up to five concatenated hairpins targeting two different genes are efficiently expressed and able to knock down their respective targets. Transplantation of hematopoietic stem cells containing a CCR5 knockdown SMIG demonstrated a sustained *in vivo* efficacy of our approach. In summary, we have defined features that optimize SMIG efficiency. Based on these results, optimized knockdown of genes of interest, such as the HIV co-receptor CCR5 and the NADPH oxidase subunit p22^{phox}, was achieved.

INTRODUCTION

The discovery and characterization of microRNA (miRNA) genes and their regulatory mechanisms not only provided a novel understanding of physiological regulation of gene expression,[1] but also opened new possibilities for miRNA-based therapeutics.[2] The centerpiece of miRNA genes is a hairpin that ultimately will give rise to a ribonucleoprotein complex,[3] which knocks down expression of target genes through identification and destruction of its transcript.[4] Structural elements of the hairpin provide a signal for processing by DROSHA and DICER,[5] leading to formation of an ~20–23 bp mature miRNA duplex.[1] The functional strand of mature miRNA duplex is incorporated into the RNA-induced silencing complex (RISC) complex,[3] which facilitates target mRNA recognition and eventually gene knockdown.[4] Synthetic miRNAs as well as by-products of the miRNA pathway, such as short hairpin RNAs (shRNAs) and small interfering RNAs (siRNAs), are now commonly used tools in molecular biology. However, the pathway has not lived up to its therapeutic potential.[2,6] siRNAs are most advanced in clinics[6]; however, they are short-lived *in vivo*, and their transient effect would require repeated *in vivo* delivery for efficient long-term gene correction.[7] shRNAs, which bypass DROSHA processing, may overload the cytoplasm with double-stranded RNA and hence lead to toxicity by obstructing the natural miRNA pathway.[8,9] Synthetic miRNAs mimic the natural pathway and should therefore overcome the above limitations,[10] but their use might be limited because of a relatively weak knockdown activity of miRNA, as compared with shRNAs.[11]

Lentiviral vectors can be used to express synthetic miRNA genes because genomic integrations of the transgene and long-term expression in recipient cells have to date been shown to be safe in patients.[12,13] However, further research is needed to optimize knockdown by synthetic miRNA genes to the extent that allows efficient therapeutic correction of pathological gene expression.

The architecture of synthetic miRNA genes, including the tridimensional structure of the hairpin, is of crucial importance for the knockdown efficiency.[14,15] In a previous study, we demonstrated that the length of the lower stem is crucial for efficient processing by

Received 6 June 2017; accepted 1 December 2018;
https://doi.org/10.1016/j.omtn.2018.12.004.

Correspondence: Francis Rousset, Department of Pathology and Immunology, Faculty of Medicine, University of Geneva, 1 rue Michel Servet, 1211 Geneva, Switzerland.
E-mail: francis.rousset@unige.ch
Correspondence: Karl Heinz Krause, Department of Pathology and Immunology, Faculty of Medicine, University of Geneva, 1 rue Michel Servet, 1211 Geneva, Switzerland.
E-mail: karl-heinz.krause@unige.ch

This is an open access article under the CC BY license (http://creativecommons.org/licenses/by/4.0/).

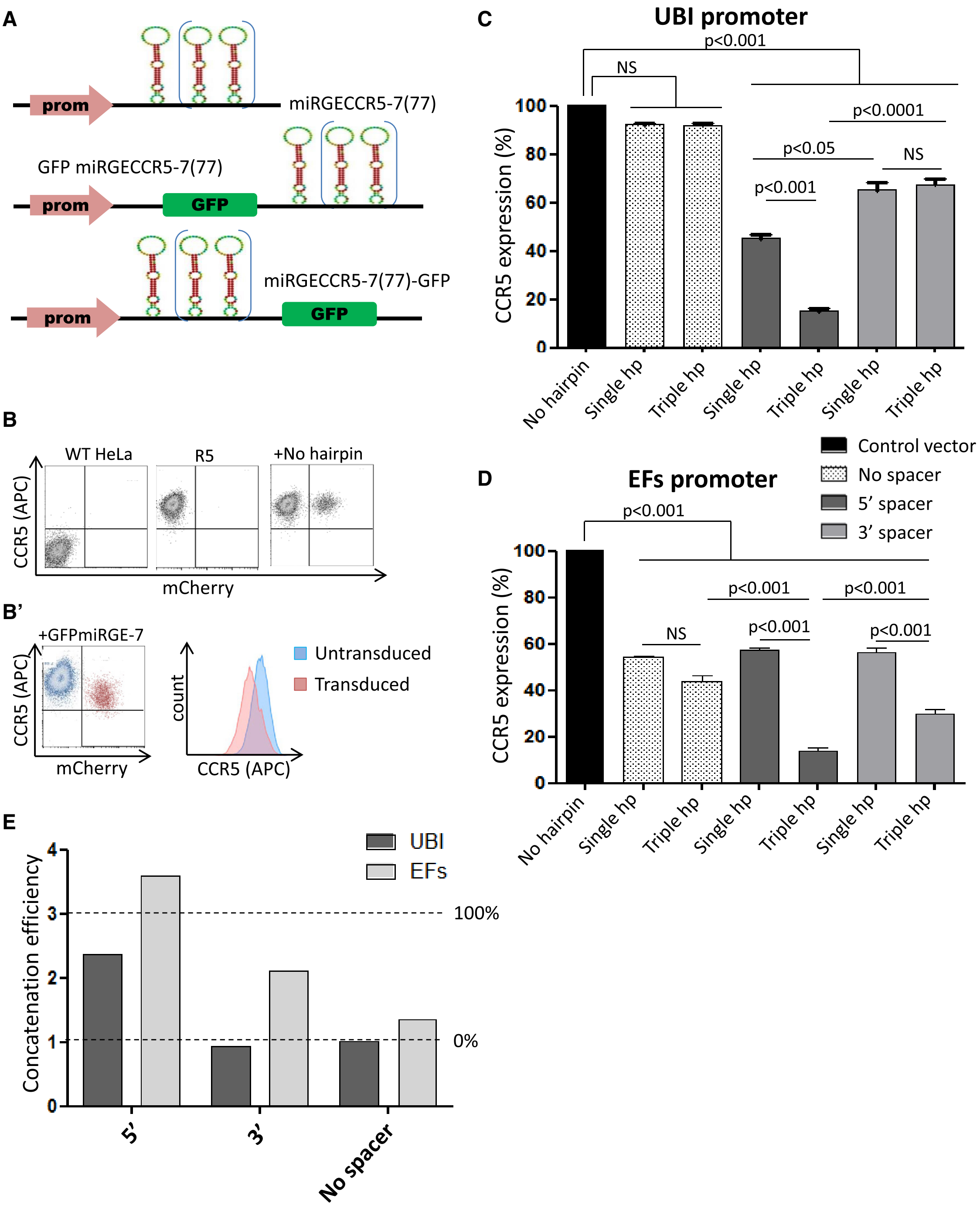

Figure 1. Effect of a Spacer Sequence on Polymerase II Promoter-Driven miRNA Knockdown

(A) Schematic representation of miRNA-based minigenes used in this experiment. All hairpins were designed to target CCR5. Two different polymerase II-dependent promoters (ubiquitin C and elongation factor 1 short) drive miRGE expression (single or triple hairpin) with or without the GFP sequence as spacer. The position of the spacer, either in 5′ or in 3′ of the promoter, was also investigated. (B) Constructs expressed with a ubiquitin C promoter or elongation factor 1 short promoter were transduced at 0.2 MOI in HeLa cells expressing CCR5. Flow cytometry determination of CCR5 expression in the transduced population (mCherry^{+}) versus the untransduced population (mCherry^{-}): wild-type (WT) HeLa cells (double negative), HeLa R5 cells (CCR5-positive). R5 + Ctrl mCherry vector; R5 + single mirGE hairpin with GFP spacer. (B′) Mean APC fluorescence values in the transduced (red) and untransduced (blue) populations were used to calculate the miRNA-mediated CCR5 knockdown. (C) Bar graph showing the

(legend continued on next page)

DROSHA and the relative abundance of mature miRNA strands available, resulting in increased target gene knockdown. However, the architecture of the miRNA gene is not limited to the hairpin structure. Other important elements include promoters and nucleotide sequences not directly linked to the hairpin (which we will refer to as a "spacer" throughout the text). miRNA genes are most of the time driven by polymerase II-dependent promoters, which allows tissue-specific and/or inducible expression.[16–19] The presence of a spacer appears to enhance knockdown efficiency[20]; however, it is not known whether sequence length or other biophysical parameters of the spacer are of importance.

Natural miRNA genes occur in a concatenated form; their architecture consists of an arrangement of several hairpins under the control of a single promoter.[21] Such concatenation is potentially a powerful tool for biotechnology, because multiple genes may be targeted simultaneously and knockdown efficiency may be increased by employing multiple miRNAs per target gene.[22,23] To date, several concatenating strategies have been developed, including the use of natural polycistronic miRNA backbones,[21,22,24,25] the concatenation of several synthetic miRNA hairpins (e.g., derived from miR-155[15] or derived from miR-16[14]), or the use of DROSHA-independent intronic miRNA (mirtrons).[26,27] However, depending on the synthetic minigene architecture, an additive effect of miRNA concatenation when targeting the same gene is not always observed[28] or has a limited effect.[29] For example, absence of additive effects of hairpin concatenate versus the single hairpin construct was reported using a synthetic miRNA gene with the Blasticidin resistance gene as a spacer. Another study using a different miRNA gene architecture, namely a cytomegalovirus (CMV) promoter and a DsRed spacer, investigated the effect of the position of hairpin within the concatenate on the knockdown efficiency. The authors reported a loss of activity of hairpins when located beyond the fourth position in the concatenate.[29] Another interesting element of miRNA architecture is the arrangement of several hairpins under the control of a single promoter. This occurs in natural miRNAs[21,22]; however, such a concatenation is also a potentially powerful tool for biotechnology.[23]

Using a previously described miRGE miRNA hairpin design,[14] we here further investigated the structural features of synthetic miRNA minigenes (SMIGs) to efficiently achieve multi-target gene knockdown. Our results provide a first demonstration that the spacer sequence, rather than spacer length itself, is crucial for efficient miRNA expression. In addition, the spacer sequence plays an important role for concatenation efficiency. Interestingly, the knockdown potency of a spacer with a single hairpin was not predictive of its concatenation efficiency. We show that with an optimal spacer sequence, concatenates with up to five efficient hairpins can be constructed, and that different genes can be targeted from a single SMIG. We also demonstrate that using the optimized SMIG architecture, efficient knockdown of clinical relevant targets, such as CCR5 and NOX3, can be achieved.

RESULTS

A Spacer Sequence Is Required for Polymerase II Promoter-Driven miRNA-Mediated Target Gene Knockdown

It has previously been suggested that a spacer sequence, either located between the promoter and the miRNA hairpin sequences or on the 3′ end of the miRNA sequence, was able to enhance artificial miRNA-based knockdown, driven by polymerase II-dependent promoters.[20,23] Moreover, concatenation of hairpins has previously been shown to exhibit an additive effect on the target gene knockdown.[14,23] To optimize the miRGE-based knockdown and better understand the role of the spacer, we placed the GFP sequence either on the 5′ or on the 3′ end of the miRGE hairpin sequences in a lentiviral vector (Figure 1A). HeLa cells expressing CCR5 (R5 cells) were transduced at an MOI of 0.2 to reduce statistical probabilities of having more than one copy of transgene per cell (Figure 1B). Both single and a triple hairpins targeting CCR5 were used. We studied two different polymerase II-dependent promoters: the ubiquitin promoter (UBI) and a spliced version of the elongation factor 1 promoter (elongation factors).[30] When the UBI promoter was used, absence of the spacer entirely precluded CCR5 knockdown, even when three hairpins were used (Figure 1C). The efficacy of the spacer depends on its position. With the spacer between the promoter and the miRNA, an efficient CCR5 knockdown was observed with a single hairpin, which was markedly enhanced with a concatenated triple hairpin construct. When the spacer was put in the 3′ position of the miRNA gene, the single hairpin showed a decreased CCR5 knockdown efficiency, whereas the increased knockdown effect of the concatenate was entirely lost (Figures 1C and 1E). The situation was slightly different for the elongation factors promoter (Figure 1D). The efficacy of a single hairpin did not depend on the presence of a spacer, but no additive effect of the triple concatenate was observed in the absence of a spacer (Figure 1E). In contrast, with the spacer between the promoter and the hairpins, a maximal effect of the triple concatenate was achieved, whereas a less pronounced effect was observed with the spacer in 3′.

Together, the data demonstrate that a spacer sequence, preferentially located in 5′ of the miRNA, is required to drive efficient knockdown via two types of polymerase II-dependent promoters. Interestingly, the spacer is also required for additive effects of the hairpin concatenation (Figure 1E).

Efficiency of miRNA-Based Knockdown Depends on the Spacer Sequence

To further understand the role of the spacer sequence and to optimize knockdown efficiency of the vector, we assessed the potency of

relative expression of CCR5 with the UBI promoter constructs. (D) Histogram showing the relative expression of CCR5 with the EF1 short promoter constructs. (E) Concatenation efficiency (E) of the different constructs as calculated on the bar graph. If E = 1, absence of additive effect is observed with the concatemerized hairpins (efficiency is 0%). If E = 3, perfect additive effect of the hairpin is observed in the concatenate (efficiency is 100%). Data represent the mean ± SEM of three independent experiments.

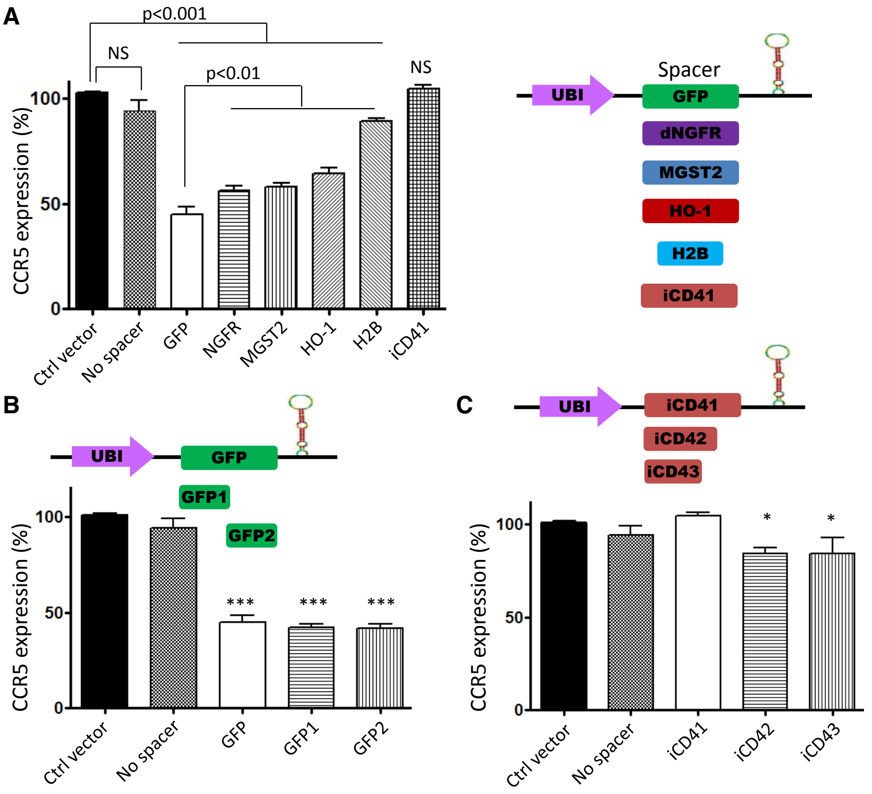

Figure 2. Efficiency of miRNA-Based Knockdown Depends on the Nucleotide Sequence of the Spacer

(A) Single miRGE hairpin-based constructs targeting CCR5 were designed with different spacers derived either from coding sequences of GFP, MGST-2 (microsomal glutathione S-transferase-2), dNGFR (truncated nerve growth factor receptor), HO-1 (heme oxygenase-1), and H2B (histone 2B), or from noncoding sequences (first intron of the CD4 gene = iCD41) and transduced at 0.2 MOI in HeLa R5 cells. Histogram showing the expression of CCR5 in the transduced population relative to the untransduced population, as assessed by FACS immunostaining. (B and C) miRGE constructs were designed with truncated forms of GFP (GFP1 and GFP2) (B) or the first intron of CD4 (iCD42 and iCD43) (C) and transduced at 0.2 MOI in HeLa R5 cells. Histograms show the level of CCR5 expression of the transduced population relative to the untransduced population. Data represent the mean ± SEM of three independent experiments.

several coding and noncoding sequences (Figure 2A; Table S1). We generated five miRGE minigenes with coding sequences as spacers: GFP, microsomal glutathione S-transferase-2 (MGST2), truncated nerve growth factor receptor (dNGFR), heme oxygenase-1 (HO-1), and histone 2B (H2B) cDNAs. We also used the first intron of the CD4 gene, iCD4, as a noncoding spacer sequence. Lentivectors carrying the respective minigenes were used to transduce HeLa R5 cells. We observed a significant knockdown of the CCR5 protein in the transduced population of cells with all coding sequences (MGST, LNGFR, HO-1, and H2B spacers). The GFP sequence spacer resulted in the highest knockdown of CCR5. The worst performing spacers were iCD4 and H2B (<10% knockdown), whereas the other coding sequences resulted in an intermediate efficiency (Figure 2A).

In an attempt to identify specific regions within spacer sequences that have an effect on the knockdown efficiency, truncated forms of GFP (GPF1, GFP2) and of the iCD4 (iCD42, iCD43) were designed (Figures 2B and 2C). Remarkably, the activity of the truncated GFP1 and GFP2 was comparable with full-length GFP (Figure 2B). The situation was different for the CD4 intron, where the shorter amplicons (iCD42 and iCD43) resulted in a moderate but significant knockdown of CCR5 (Figure 2C). However, these truncated CD4 first intron sequences were still inefficient spacers when compared with sequences of similar length (GFP1 or GFP2) (Figures 2B and 2C). These results demonstrate that the spacer activity does not simply depend on the length, but that the nucleotide sequence also seems to determine its efficiency. We did not observe a correlation between the predicted minimum free energy (MFE) of spacers and the knockdown efficiency (Figure S1A). This raises the possibility that the secondary structure of the spacer is not a crucial element. We assessed GC content of the spacer sequences and found that spacers with higher GC content tended to correlate with the higher knockdown efficiency (Figure S1B).

The Spacer Sequence Determines the Additive Effects of miRNA Hairpin Concatenation

To confirm the role of the spacer sequence in the concatenation potency of the vector, multi-hairpin constructs were designed with different spacers: GFP, GFP2, MGST2, or H2B (Figure 3A). When the GFP or GFP2 sequence was used as a spacer, the concatenation of three hairpins dramatically enhanced CCR5 knockdown compared with a single hairpin construct (from 60% to 85% CCR5 knockdown) (Figures 3B and 3C). When MGST2 was used as a spacer, substantially different results were obtained. As illustrated in Figure 2A, with a single hairpin, the MGST2 sequence had a good spacer activity, albeit not as potent as the GFP sequence. However, with MGST2 as the spacer, no additive effect of a three-hairpin concatenation was observed (concatenation efficiency close to 1) (Figure 3C). Thus, we observe a dissociation between the spacer potency with a single hairpin, as compared with the concatenation activity. Although the former is in a comparable range for GFP and MGST2, the latter is virtually absent with MGST2 as a spacer (Figure 3C). The opposite was observed with H2B as a spacer: a rather poor knockdown was observed with a single hairpin (~10%), whereas there was an improved concatenation effect as judged by the CCR5 knockdown with the triple hairpin construct (40% knockdown) (Figures 3B and 3C). To investigate whether this observation also applies to hairpins targeting genes other than CCR5, we constructed triple hairpin

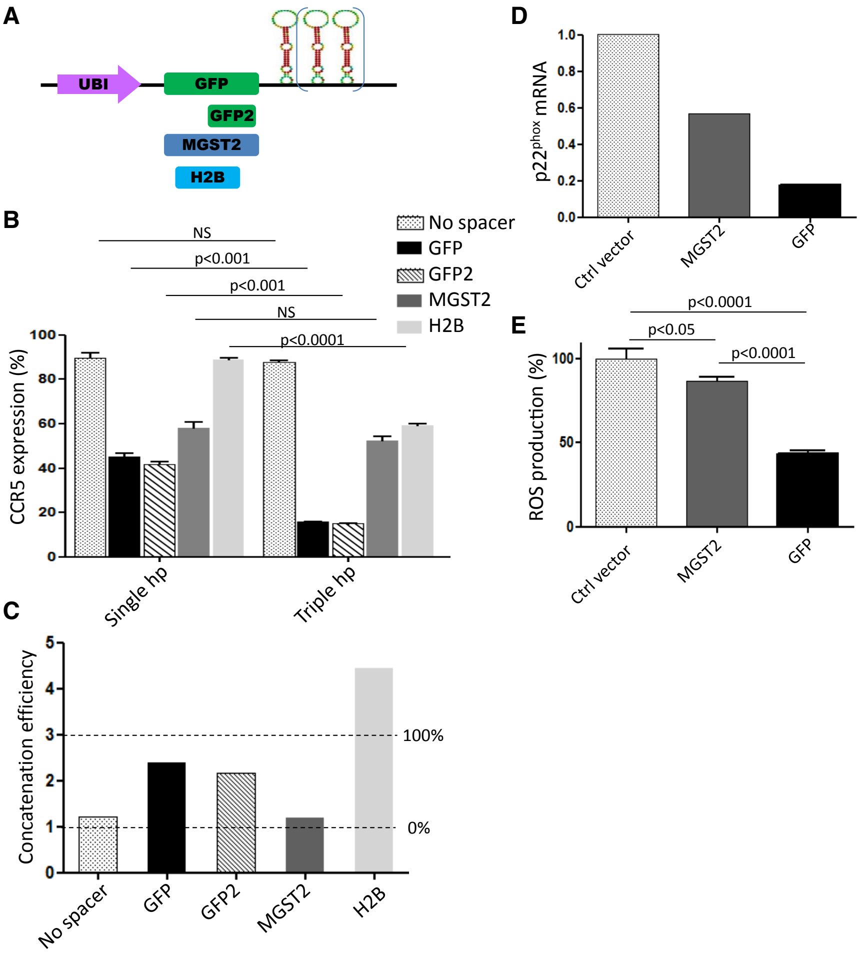

Figure 3. Efficient Concatemerization of Hairpins Depends on the Nucleotide Sequence of the Spacer

(A) The efficiency of MGST2, H2B, and GFP as spacer was assessed on miRGE hairpin concatenation (three hairpins concatenate) in HeLa-expressing CCR5 cells. (B) Histogram shows the level of CCR5 expression of the transduced population relative to the remaining untransduced population for the GFP spacer (black bars), the second part of GFP (GFP2) (hatched), MGST2 spacer (dark gray bars), and the H2B spacer (clear gray bars). (C) The bar graph shows the concatenation efficiency for the triple hairpin constructs, as calculated with the formula in Figure S3. For the calculation of the concatenation efficiency, CCR5 knockdown with a single miRGE hairpin (knockdown potency [KP]) and with a triple miRGE concatenate (concatenation potency [CP]) were considered. (D and E) The potency of MGST2 and GFP as spacers was also compared in a triple hairpin concatenate targeting the NADPH oxidase subunit $p22^{phox}$. mRNA level of $p22^{phox}$ as assessed in the promyelocytic leukemia cell line PLB985 by qPCR (D) and on NADPH oxidase activity by Amplex red assay (E). Data represent the mean ± SEM of three independent experiments.

SMIG targeting the NOX subunit $p22^{phox}$ (*CYBA*) with either MGST2 or GFP as spacer sequences (Figures 3D and 3E; see also Figure S1). These constructs were used to transduce the promyelocytic leukemia cell line PLB-985, which upon differentiation toward a neutrophil-like phenotype expresses all phagocyte NADPH oxidase subunits (including NOX2 and $p22^{phox}$/CYBA) and produces reactive oxygen species (ROS) through this NADPH oxidase. We observed a 50% decrease in the CYBA mRNA level using the MGST2 spacer-triple hairpin constructs, whereas with a GFP spacer CYBA mRNA knockdown was >80% (Figure 3D). These results corroborate our previous observation with hairpins targeting CCR5. We also looked at functional activity of the phagocyte NADPH oxidase, namely ROS generation (Figure 3E). The production of ROS was inhibited by 60% with the GFP-triple hairpin concatenate targeting $p22^{phox}$. In contrast, by replacing GFP with MGST2 as a spacer, ROS production was inhibited by no more than 20%. These data confirm that, although efficient knockdown is seen with a single hairpin, the MGST2 spacer has poor concatenation activity (Figure 3C; Figure S2). These experiments demonstrate that the spacer sequence is not only required for the knockdown efficiency with a single hairpin but is also required for the additive concatenation effect: the concatenation potency. Thus far, the GFP spacer exhibited the most effective combination of knockdown and concatenation (Figures 3B and 3C).

Translation-Independent Activity of the GFP Spacer in Cell Lines and Tissue Explants

Among the tested candidates, the GFP sequence was most efficient as a spacer, both with respect to knockdown potency with a single hairpin and concatenation potency. Other coding sequences also yielded some significant knockdown activity, while the CD4 intron was inactive. To test whether protein translation of GFP was required for optimal functioning of the SMIG, we designed a construct harboring stop codons in each possible reading frame (Figure 4A). As predicted, no fluorescence was detected in the cells transduced with stopGFP (Figure 4B). The CCR5 knockdown achieved with the stopGFP spacer was comparable with knockdown with the standard GFP spacer (~50% with a single hairpin construct). These results are relevant for two reasons: (1) they refute the hypothesis that protein translation of the spacer is important for the function of SMIG; and (2) they provide highly efficient spacer, which does not lead to translation of the xenogene GFP and is therefore compatible with a future clinical use.

To demonstrate the therapeutic potential of the optimized SMIG including the stopGFP spacer, we investigated a potential clinical application, namely knockdown of the inner ear NADPH oxidase NOX3. This ROS-producing NADPH oxidase has been shown to be a relevant source of ROS leading to inner ear damage, and it is hence an attractive knockdown target for inner ear protection.[31]

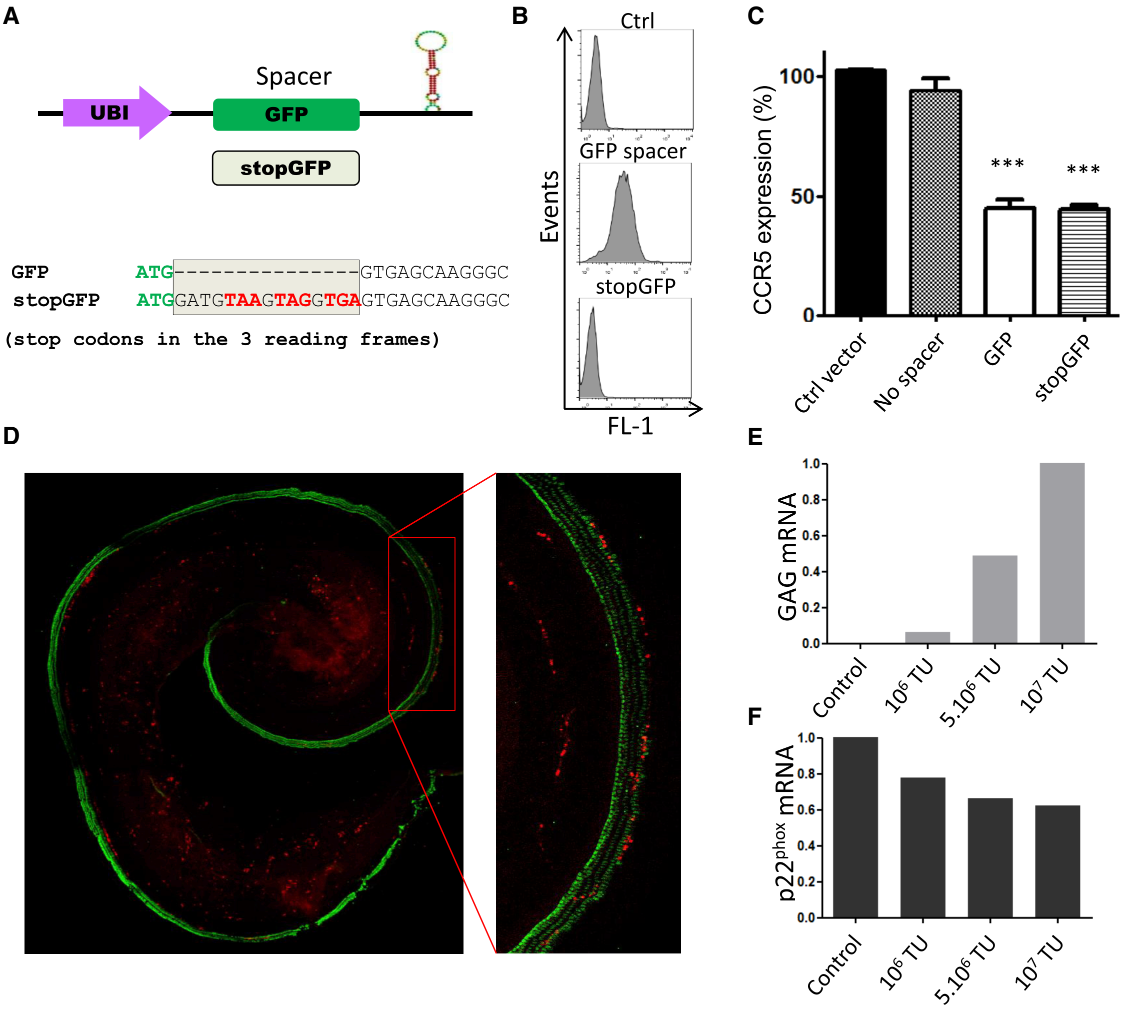

Figure 4. Untranslated Spacer Supports miRNA Minigene Architecture and Knockdown of Inner Ear Target Genes

(A) Design of the stopGFP spacer, harboring stop codons in all possible reading frames of the GFP cDNA in 3′ of the initiation codon (ATG). (B) HeLa R5 cells were transduced at >1 MOI with both coding and noncoding forms of GFP. FACS histograms show the fluorescence of GFP in transduced cells. The GFP fluorescence of cells transduced with the stopGFP construct was comparable with the control (non-transduced HeLa cells). (C) The spacer activity of stopGFP sequence was also assessed by FACS on CCR5 expression after 0.2 MOI transduction in HeLa R5 cells. The histogram shows the expression of CCR5 of the transduced population relative to the untransduced population as assessed by FACS immunostaining. Data represent the mean ± SEM of three independent experiments. (D) Organotypic culture of newborn rat cochlear explants transduced with increasing amounts of stopGFP triple miRGE hairpin concatenate targeting p22^{phox} (10^6–10^7 vector particles). Hair cells are stained for myosin 7a (in green) and efficiently transduced cells express the marker gene mCherry (red). After 5 days *in vitro*, expression of the viral gene GAG (E) and p22^{phox} (F) was assessed by qPCR. TU, transducing units.

For this purpose, we designed a triple miRGE concatenate, targeting the NOX3 subunit p22^{phox}, under the control of the UBI promoter, and with stopGFP as a spacer. To identify transduced cells, the mCherry coding sequence under the control of the phosphoglycerate kinase 1 (PGK) promoter was also included in the construct. Using this construct, we transduced newborn rat cochlear explants (Figure 4D). Using RT-PCR to detect and quantify the lentiviral GAG gene, we confirmed a dose response for vector transduction (Figure 4E). Transduced cells could also be identified by mCherry red fluorescence (Figure 4D; green fluorescence is a marker for hair cells). As seen in this picture, only a minority of hair cells were transduced with the vector under our experimental conditions. Despite this suboptimal transduction efficiency, results showed a dose-dependent decrease in p22^{phox} mRNA, confirming the efficiency of the miRGE vector with a second clinically relevant target gene (Figure 4F).

Sustained miRNA-Mediated Knockdown of CCR5 in Circulating Leukocytes Derived from Human Hematopoietic Stem Cells

To further demonstrate the *in vivo* efficacy and the therapeutic potential of the optimized SMIG including the stopGFP spacer, we investigated another promising clinical application, namely

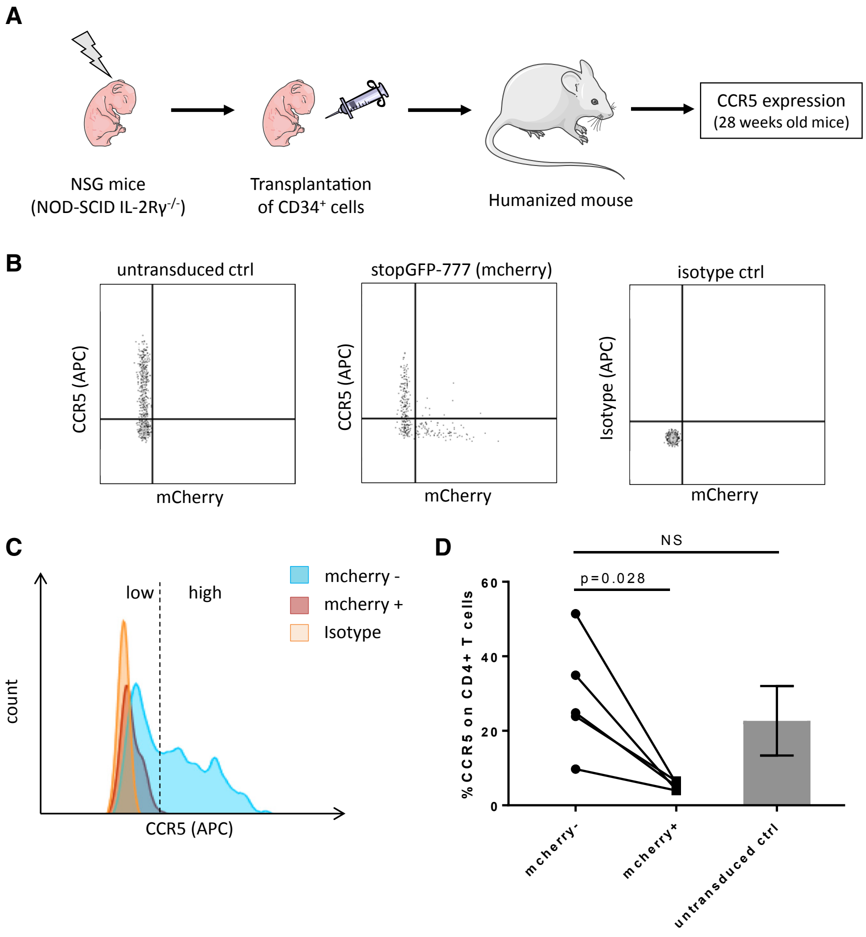

Figure 5. Optimized Minigene Architecture Allows Sustained *In Vivo* Knockdown of CCR5 in Circulating Cells

(A) Six NGS newborn mice were engrafted with 260,000 human CD34+ hematopoietic stem cells (HSCs), following transduction with triple hairpin concatenate targeting CCR5. At the age of 28 weeks, blood was harvested for analysis of CCR5 expression. (B) FACS plots showing CD4+ T cells from mice transplanted with non-transduced HSC (left) or transplanted with stopGFP-777 transduced HSC (center). Right plot shows CD4+ T cells stained with irrelevant antibody (untransduced HSC). mCherry+ cells indicate effectively transduced cells. (C) Histogram showing expression level of CCR5 in transduced (mCherry+) and remaining untransduced (mCherry−) CD4 T cell population for a single transplanted mouse, relative to irrelevant antibody (isotype). Cells on the right of the dotted line represent high CCR5-expressing CD4 T cells. (D) Comparison of the CCR5 expression level in high CCR5 CD4+ T cells population in five engrafted mice. Untransduced ctrl stands for mice engrafted with non-transduced human CD34+ cells (n = 3).

knockdown of the HIV co-receptor CCR5 *in vivo* (Figure 5). For this purpose, we transduced human CD34+ hematopoietic stem cells (HSCs) with a triple miRGE concatenate, targeting CCR5, under the control of an elongation factors promoter, and with stopGFP as a spacer. To identify transduced cells, the mCherry coding sequence under the control of the PGK promoter was also included in the construct. After transduction, HSCs were engrafted in NGS (NOD scid gamma) mice following irradiation (Figure 5A), achieving an engraftment rate varying between 12.4% and 44%, after 23 weeks (Figure S4). 28 weeks following the engraftment, CCR5 expression was investigated in the circulating blood (Figure 5; Figure S5). The results revealed two kinds of CD4+ T cells with respect to CCR5 expression in untransduced control and mCherry-negative cells (Figures 5B and 5C). The proportion of high CCR5-expressing CD4 T cells varied from less than 10% to more than 50% with an average close to 25% in five of the six engrafted animals (Figure 5D, see mCherry and untransduced ctrl). Note that in one of the six engrafted animals, the high CCR5 CD4 T cells population was virtually absent and therefore not taken into account in Figure 5D. Importantly, we observed a dramatic decrease of the CCR5 expression level in the mCherry+ transduced population (Figure 5D, mCherry+).

The Spacer Sequence Regulates the Steady-State Levels, but Not the Half-Life of miRGE

To better understand the mechanisms of spacer activity, we designed PCR primers to quantify unprocessed miRGE hairpins (pri-miRGE) (Figure 6A) or the mature miRGE (Figure 6E). As seen in Figures 6B and 6C, relative expression of the miRGE pri-miRNA was significantly stronger in cells transduced with the stopGFP spacer than in the cells transduced with the MGST-2 or NGFR-based vector. Note that miRGE expression was below the detection threshold with the CD4 first intron as spacer, as also seen in the absence of a spacer. To investigate whether this increase in the steady-state levels of miRGE was due to a prolonged half-life of the transcript, HeLa R5 cells were treated with actinomycin D for different time periods to block transcription (Figure 6D). mRNA was harvested and miRGE expression levels assessed by qPCR of the pri-miRGE at the different time points. Results showed an estimated mirGE half-life of approximately 30 min with the stopGFP spacer (Figure 6D), similar to that seen with the NGFR and MGST spacers. The steady-state level of the mature miRGE demonstrated that stopGFP spacer allows the best expression of the mature miRGE (Figure 6F). This observation was also valid when comparing stopGFP and MGST2 spacers with triple hairpin concatenates (Figure 6G). Interestingly, both levels of precursor and mature miRGE were similarly impacted by the spacer sequence. These results strongly suggest that spacer activity is not linked to stability of miRNA transcript nor processing. Our results would rather be compatible with a mechanism where the spacer is relevant for the transcription of the SMIG.

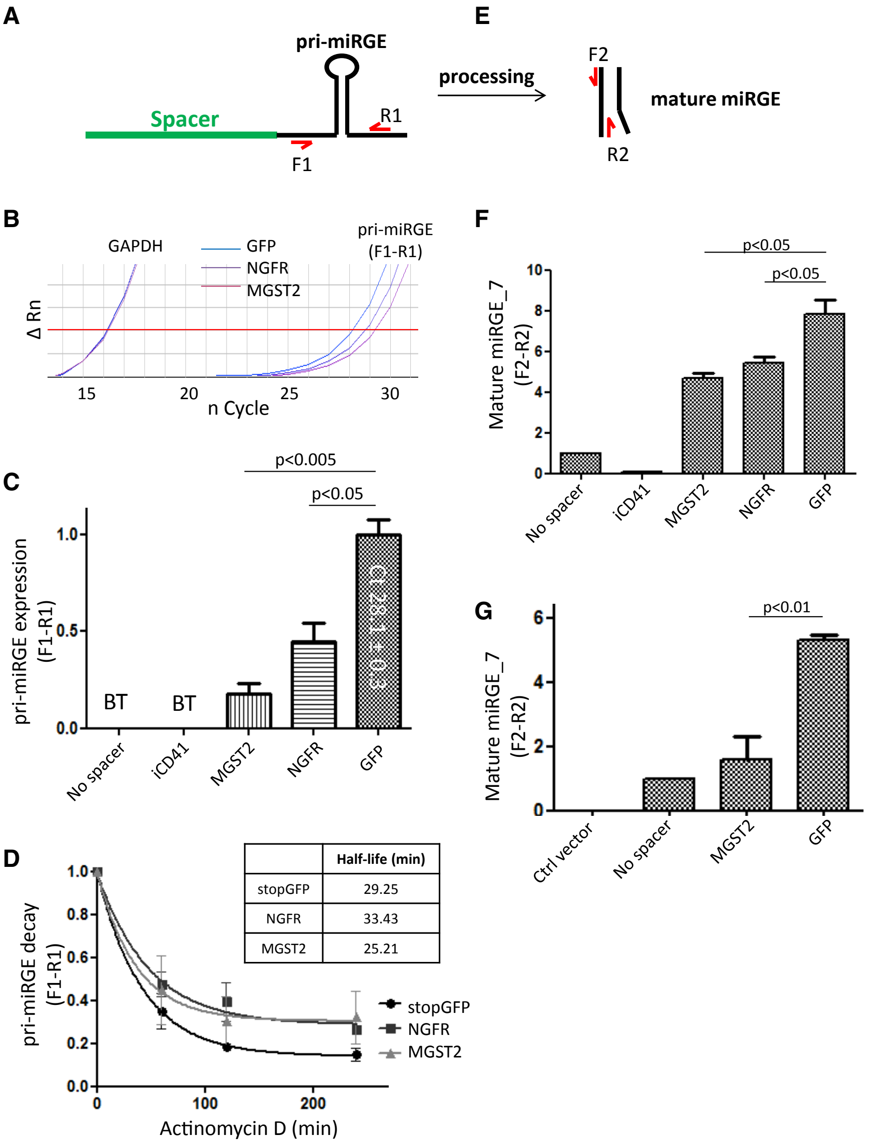

Figure 6. The Spacer Sequence Regulates the Steady-State Levels of Mature miRNA

HeLa cells expressing CCR5 were transduced with single hairpin vector with GFP, LNGFR, MGST2, or iCD41 as spacers. (A) Unprocessed (pri-miRGE) or (E) mature miRGE level was assessed by qPCR using primers matching the flanking region of pri-miRGE (F1-R1) or the targeting strand of the mature miRGE (F2-R2). (B) Representative qPCR amplification plot of unprocessed miRGE level with GFP, NGFR, and MGST2 as spacers. Note that without spacer or with iCD41 as spacer, miRGE level was below detection threshold. GAPDH was used as housekeeping gene. ΔRn of 0.2 was defined as the threshold (red line). (C) Bar graph shows the relative level of unprocessed miRGE as averaged from three independent experiments. The highest value of miRGE expression, normalized to 1.0, corresponds to a cycle threshold (Ct) value of 28.1 ± 0.3. (D) Transcription was blocked with actinomycin D at different time points (0–240 min), and effect of the spacer was assessed on unprocessed miRGE half-life. Graph shows the relative miRGE decay over time, and half-life for each spacer is displayed in the table. (E–G) Comparison of the steady-state level of the mature miRGE (E) as assessed by qPCR from HeLa cells transduced with single hairpin (F) or triple hairpin concatenates (G) with different spacers. Data represent the mean ± SEM of three independent experiments. BT, below detection threshold.

Maximizing Concatenation and Achieving Multi-target Gene Knockdown

The concept of successive cloning of miRNA hairpins was previously demonstrated with a CMV promoter-mediated miRNA-based lentivector.[23] Sun et al.[23] successively cloned up to three miRNA hairpins, expressed in a lentivector system with a single CMV promoter, and reported that knockdown of the target gene was proportional to the number of hairpins. We previously confirmed these observations when using a UBI promoter to drive expression of up to three miRGE hairpins designed to target CCR5.[14] To further investigate the possibility of a multi-target gene knockdown vector with a single promoter-driven miRNA cluster, a fourth and a fifth mirGE hairpin, either targeting CCR5 or a second target gene (GFP in this case), was added to the triple CCR5 construct (Figure 7A). Concatenation of the hairpins led to a significant increase of the mature miRGE steady-state level as a function of the number of hairpins present in the concatenate (Figure 7B). Interestingly, the addition of a fourth hairpin targeting CCR5, while leading to the highest mature miRGE level, did not provide additional decrease in CCR5 expression compared with the triple hairpin construct, arguing for a possible saturation of the CCR5 target sites with the miRGE (Figure 7C). On the other hand, when we replaced the fourth hairpin with a hairpin targeting GFP, not only did CCR5 knockdown remain at its maximum level (~90%), but there was also a significant decrease in GFP fluorescence (Figure 7C). Thus, while the hairpin in the fourth position did not further enhance CCR5 knockdown, it was clearly still efficiently processed, as witnessed by the GFP knockdown (Figure 7D) and the mature miRGE-GFP steady-state level (Figure 6E). Interestingly, a fifth hairpin targeting GFP displayed similar knockdown efficiency and level of mature miRGE as the fourth, still without affecting knockdown of the CCR5. More importantly, miRGE_GFP steady-state levels as well as GFP knockdown mediated by the fourth or the fifth miRGE hairpins was comparable with knockdown achieved with a single miRGE hairpin targeting GFP. Thus, with UBI as promoter and stopGFP as spacer, there was no loss of activity with up to five concatenated hairpins. However, the efficiency of the five-hairpin concatenation strongly depended on the spacer. Indeed, the use of

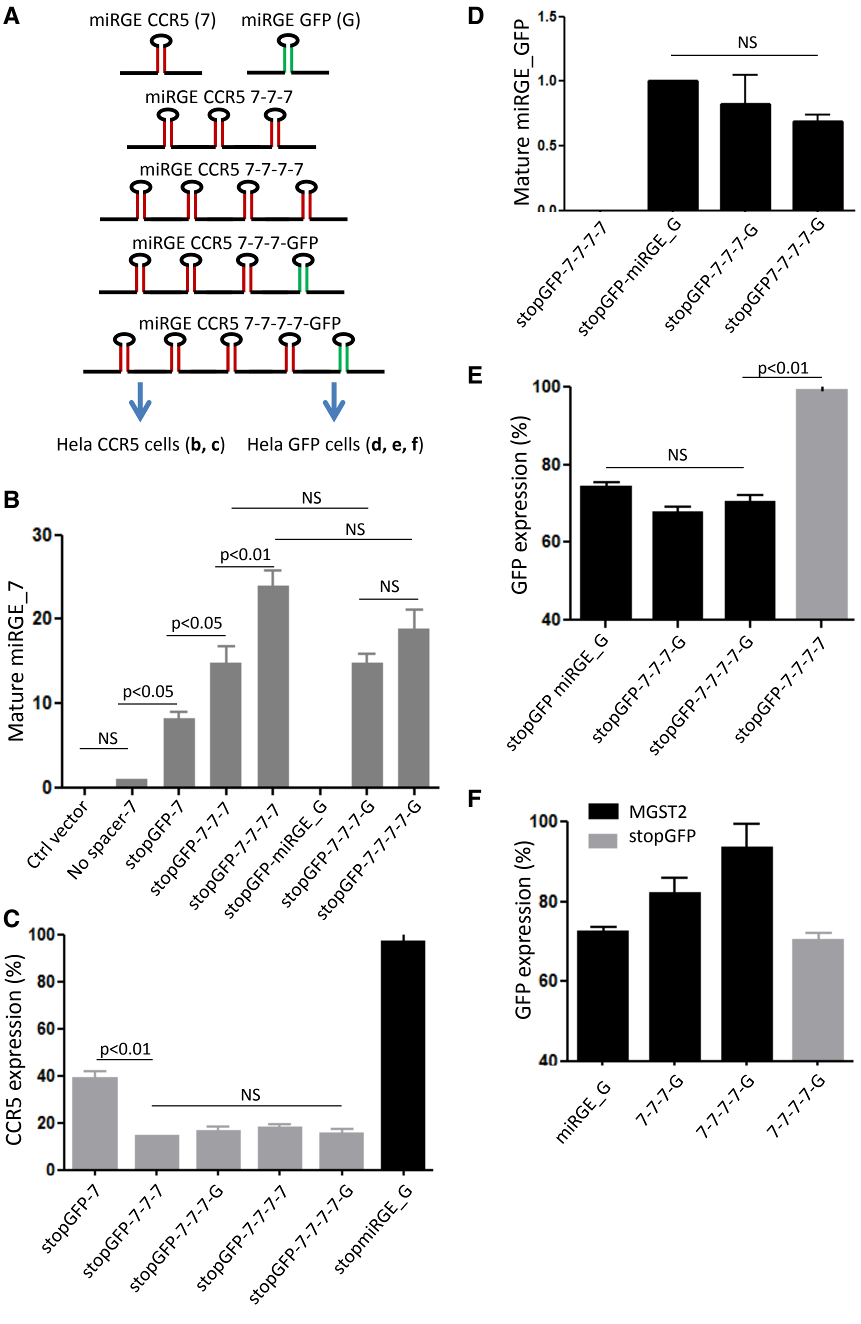

Figure 7. Optimized miRNA Minigene Architecture Allows Maximal Hairpin Concatenation and Efficient Multi-target Knockdown

(A) Efficiency of the stopGFP triple concatenate targeting CCR5 (mirGE 7-7-7) was compared with constructs harboring a fourth or a fifth hairpin either targeting CCR5 or a second target: GFP. Bar graphs show the steady-state level of the mature miRGE targeting CCR5 (7) (B) or targeting GFP (G) (D), as assessed by qPCR and the ability of these constructs to knock down CCR5 expression (C) or GFP expression (E) in HeLa cells. Vectors expressing a single miRGE hairpin targeting CCR5 or GFP were used as controls. (F) When replacing the stopGFP with MGST2 as spacer, activity of the fourth and the fifth hairpin was decreased, as compared with the single miRGE hairpin control (miRGFP) or the fifth hairpin of the stopGFP construct. Data represent the mean ± SEM of three independent experiments.

MGST2 as spacer led to a dramatic decrease of the fourth and fifth hairpin GFP knockdown potency (Figure 7F). These data demonstrate that optimized SMIG architecture allows for efficient multi-target gene knockdown upon a single promoter-driven, multi-hairpin construct.

DISCUSSION

We report in this study the development and optimization of SMIGs for therapeutic gene knockdown. We analyzed knockdown efficiency by single hairpins as well as the concatenation potential. For both features, the insertion of a spacer within the SMIG was crucial. Nucleotide sequence, rather than length of the spacer, was a key feature for optimized knockdown activity. The spacer determined miRNA steady-state levels, rather than transcript stability. The relevance of these modifications was confirmed by *in vivo* experiments demonstrating sustained miRNA-mediated knockdown of CCR5 in HSC-derived leukocytes.

Although the expression of very short RNAs, such as shRNAs, is usually driven by polymerase III-dependent promoters, the expression miRNAs, either natural or synthetic, is usually driven through polymerase II promoters.[16] Polymerase III-driven shRNA expression leads to very high transcript levels[32] and is associated with saturation of the miRNA processing pathway leading to decreased levels of processed endogenous miRNAs and various toxicities.[9] These problems have not been reported with polymerase II-dependent expression of SMIG. Another advantage of polymerase II-dependent promoters is the possibility for tissue-specific and inducible expression.[17,19,33] However, the main limitation of previously described synthetic miRNAs was the relatively low knockdown efficiency. In our study, we compared two different polymerase II-dependent promoters, namely the UBI and the EF1 short promoter. Although the two promoters impacted differently on SMIG knockdown efficacy (see below), in general, most of the results described below are valid when using either promoter.

One of the key findings of our study is the complex role of a so-called spacer. The need for a spacer in the SMIG was first suggested by Stegmeier et al.[20] (see also Introduction). As already implied by the

term "spacer," the initial concept was the need for a spatial separation between the promoter and the miRNA hairpins; however, constructs with a spacer on 3′ of the hairpin have also been described.[23] Our study clearly demonstrates that spacer activity is most efficient when placed between the promoter and hairpin. Importantly, however, our data refute the concept that the spacer is simply a spatial separator. Indeed, as shown in Figure 2, the length of the spacer does not predict its efficiency. Whereas the GFP coding sequence provided a high-level knockdown, a CD4 intronic sequence led to poor miRNA expression and therefore knockdown efficiency. The apparent superiority of coding sequences over the intronic sequence might suggest that translation of the coding sequence was required for spacer activity; however, this was also refuted by the experiments with the stopGFP construct (Figure 4). Another possible explanation would be an impact of the spacer on the stability of the miRGE transcript. Our results do not favor this hypothesis. Indeed, we show that the spacer sequence enhances the steady-state level of miRGE without any effects on its half-life (Figure 5). Thus, the spacer most likely impacts transcription of the miRNA hairpin. The predicted MFE of the respective spacer sequences was not correlated with miRNA efficiency and knockdown, suggesting that the secondary structure of the transcript is not crucial (Figure S1A). It is possible that certain biophysical properties of the spacer are of importance; indeed, there was a tendency (albeit not statistically significant) for a correlation between the spacer GC content and SMIG knockdown efficiency (Figure S1B). It is, however, also worth considering the possibility that spacer nucleotide sequence might be of importance. Certain nucleotide motifs could enhance or limit miRNA transcription and thereby steady-state levels of the SMIG transcript. Indeed, genes such as human BRCA1,[34] mouse c-*fos*,[35] or human c-Myb[36] exhibit transcription repression motifs within their first intron sequence; such a mechanism might account for annihilation of SMIG knockdown when using the CD4 intron as spacer. Note also that inhibition of miRNA activity by an intronic sequence has been reported previously.[37] Thus, one might formulate a working hypothesis, where biophysical properties (e.g., GC content) optimize spacer activity, and where sequence motifs (e.g., found in intronic regions) lead to an inhibitory effect. Such a theory, although still speculative, would be supported by our spacer truncation experiments. Whereas truncated forms of efficient spacer GFP exhibited the same knockdown potency as the full-length variant, truncation of the "inhibitory" CD4 intron spacer led to a partial recovery of SMIG activity (Figure 2C).

Another key finding of our study is the dissociation of the impact of SMIG design on single hairpin knockdown efficiency versus concatenation potential. For example, in the absence of a spacer, the elongation factors promoter provided an acceptable single hairpin knockdown; however, concatenation potential was entirely lost under these conditions. Similarly, the use of the MGST2 spacer within an SMIG provided an acceptable single hairpin knockdown, but led to a loss of concatenation potential (Figure 3C). On the other hand, despite rather poor knockdown with a single hairpin construct, the H2B spacer sequence allowed efficient concatenation of up to three hairpins (Figure 3D). Although our studies are the first analysis comparing single hairpin knockdown with concatenation potential, certain previous studies retrospectively corroborate our results. For instance, when using the Blasticidin resistance gene as a spacer for miRNA, no concatenation potential was observed.[28] Hu et al.[29] reported no spacer requirement for the efficiency and concatenation potential of a CMV promoter-driven SMIG. This is in contradiction with other studies using the CMV promoter.[20,23] We think that this is most likely explained by the fact that the study by Hu et al.[29] used large copy number transfection of the miRNA, which could mask the spacer requirement when target cells are transduced. Indeed, all studies using CMV promoter-driven SMIG with a low copy number vector transduction have consistently reported the use of spacer sequences.[20,23] In our study, the stopGFP spacer allowed concatenation of up to five hairpins without loss of activity of any of the hairpins. Interestingly, maximum CCR5 knockdown required only three miRGE hairpins. This suggests that the endogenous miRNA pathway, including processing by DROSHA, DICER, EXPORTIN5, and RISC, is not oversaturated with CCR5 hairpins, but rather that CCR5 reaches a background expression level and cannot be further knocked down. Note that the potency of the different knockdown sequences was markedly different. Although a single CCR5 knockdown hairpin could achieve up to a 50% decrease in CCR5, the single GFP knockdown hairpin decreased GFP expression at best by 20%. These different knockdown efficiencies were not related to SMIG architecture, but simply to the efficiency of the targeting sequence.

In summary, we describe an optimized architecture for an SMIG. We demonstrate that miRNA expression from at least two different polymerase II-dependent promoters is highly dependent on the presence of a spacer sequence, preferably located on the 5′ of the miRNA. The spacer sequence enhances miRNA steady-state levels without a detectable impact on RNA stability. The biophysical properties and/or sequence of the spacer, rather than its length or its secondary structure, seem to be of crucial importance. The expression of up to five hairpins can be achieved with this system through concatenation, allowing efficient targeting of several genes. This multi-target gene knockdown feature of optimized SMIG constructs is of major therapeutic interest. We also demonstrate that the use of our system is not limited to cultured cell lines. We achieve efficient gene knockdown in a complex organ such as the cochlea, as well as *in vivo* in leukocytes derived from transplanted HSCs. Thus, our results not only provide novel insights into miRNA expression systems, but also provide novel tools for the investigation of functional genomics and for therapeutic applications.

MATERIALS AND METHODS

All animal experimental procedures were conducted under approved protocol of the local veterinary office, authorization number ZH181/17 and BE 124/13.

Construction of miRNA-Containing Plasmids and Lentiviral Vectors

The plasmids were constructed using the gateway system as described previously.[14] With the exception of MGST2, spacer sequences were

amplified by PCR using Herculase II polymerase (Agilent, Santa Clara, CA, USA) with forward and reverse primers carrying, respectively, EcoRI and XhoI restriction sites and cloned into a pENTR vector (Invitrogen) by digestion and ligation steps (Table S2). Most of the primers used for the cloning of spacers were designed with AttB1 (forward primer) and AttB2 (reverse primer) recombination sites at the 5′ extremity (Table S4). The MGST2 spacer was obtained from pOTB7-MGST2 plasmid (transomic) by EcoRI/XhoI restriction digestion and subsequent ligation into the pENTR vector. mirGE hairpins were amplified using the same strategy and forward and reverse primers carrying, respectively, SpeI and BamHI restriction sites. miRGE hairpin concatenates were made using different couples of restriction enzymes on the miRGE primers or by blunt ligation as in Sun et al.[23] Each new miRGE addition was verified by sequencing the pENTR vector. The amplicon parts of each clone, including spacer and miRGE hairpins, were systematically verified by sequencing. The oligos for the miRGE PCR template and primers were obtained from Microsynth (Balgach, Switzerland). miRGE hairpin template sequences targeting CCR5, GFP, and $p22^{phox}$ are available in Table S3. The final lentivector plasmid was generated by an LR Clonase II (Invitrogen, Carlsbad, CA, USA)-mediated recombination of a pENTR plasmid containing the human UBI promoter (pENTR-L4-UBI-L1R) or the elongation factor 1 short promoter (pENTR-L4-EFs-L1R) and a lentivector destination cassette (pCWX-R4dEST-R2-PC) containing an additional transcription unit encoding for mCherry marker gene upon human PGK promoter. The GFP target sequence, 5′-AAGAACGGCATCAAGGTGAACT-3′, was taken from a previous publication.[38] The human CCR5 (GenBank: NM_000579.3) target sequence (T7) 5′-aAGTGTCAAGTCCAATC TATGA-3′ was previously used.[14]

Lentiviral Vector Production and Titration

Lentiviral vector stocks were generated using transient transfection of HEK293T cells with the specific lentivector transfer plasmid, the psPAX2 plasmid encoding gag/pol, and the pCAG-VSVG envelope plasmid, as previously described.[17,18] Lentivector titration was performed using transduction of HT-1080 cells followed by flow cytometry quantification of $mCherry^+$ cells 5 days after transduction, as previously described.[17,18]

Cell Culture and Knockdown Analysis

All cell lines were cultured in high-glucose DMEM (Sigma) supplemented with 10% fetal calf serum, 1% penicillin, 1% streptomycin, and 1% L-glutamine. For each knockdown assay, cells were analyzed at least 5 days after transduction. For CCR5 knockdown studies, a subclone of HeLa-derived TZMbl cells (AIDS Repository, Germantown, MD, USA), expressing high levels of human CCR5, named here HeLa R5, was used. For GFP knockdown, the same cells were used after GFP transduction at one copy of the vector and sorting of the GFP-positive cells. CCR5 expression was detected using an anti-human CCR5-allophycocyanin (APC) antibody (Cat. 550856; BD Pharmingen) and flow cytometry analysis using fluorescence-activated cell sorting (FACS) Cyan (Beckman Coulter). GFP expression was assessed on the same flow cytometer using GFP fluorescence median. In brief, HeLa cells were transduced at 0.2 MOI with the miRGE-based knockdown vector to avoid the presence of a high copy number of the vector per cell and to obtain comparable conditions. GFP or CCR5 expression was compared between the transduced and the remaining untransduced population of cells and expressed as a percentage of CCR5 expression relative to the untransduced population.

Real-Time qPCR

Cells or organotypic explant of organ of Corti were harvested and mRNA was extracted using QIAGEN RNeasy mini kit following the manufacturer's instructions. RNA concentration was determined using a NanoDrop. 500 ng was used for cDNA synthesis using Takara PrimeScript RT reagent Kit following manufacturer's instruction. Real-time PCR was performed using SYBR green assay on a 7900HT SDS system from ABI. The efficiency of each primer was verified with serial dilutions of cDNA. Relative expression levels were calculated by normalization to the geometric mean of the two housekeeping genes GAPDH and EF1a and the GAG lentivector gene. The highest normalized relative quantity was arbitrarily designated as a value of 1.0. Fold changes were calculated from the quotient of means of these normalized quantities and reported as ±SEM. Sequences of the primers used are provided in Table S1.

Real-Time qPCR for Mature miRNA Detection

HeLa R5 cells were transduced at 0.2 MOI with lentivectors carrying the different SMIGs. Transduced population (expressing mCherry) was sorted by FACS resulting in a homogeneous cell population carrying a single copy of the vector per cell. Total RNA was extracted using TRIzol Reagent (Ambion) according to the manufacturer's instructions. RNA concentration was determined using a NanoDrop. One hundred nanograms of RNA was used for the reverse transcription (miRCURY locked nucleic acid [LNA] miRNA PCR, polyadenylation and cDNA synthesis kit [exiqon]). Reverse transcription was followed by real-time PCR amplification (ExiLENT SYBR Green master mix kit [exiqon]) with LNA-enhanced primers. Relative expression levels of the mature miRGE were calculated by normalization to the geometric mean of the two housekeeping miRNA (U6 and RNU5G). Fold changes were calculated from the quotient of means of these normalized quantities and reported as ± SEM. Sequences of the LNA-enhanced primers were not provided by the manufacturer.

ROS Measurement by Amplex Red Assay

PLB-985 cells were cultured in RPMI medium (GIBCO), transduced as described above, and differentiated into neutrophil-like cells during 5 days in the presence of 1.25% DMSO. Levels of H2O2 produced by intact PLB-985 cells after stimulation of NOX2 with 100 nM phorbol myristate acetate (PMA) were then measured using Amplex Red fluorescence as previously described.[39] Fluorescence was measured with a FluoSTAR OPTIMA, BMG LABTECH instrument at 37°C.

Organotypic Culture and Transduction of Rat Organ of Corti

Three-day-old Wistar rats were decapitated and the heads were cut sagittally to remove the brain. The two otic capsules were isolated

and transferred into ice-cold Hank's balanced salt solution (HBSS) (Invitrogen, USA) for sterile dissection under a binocular microscope (Nikon SMZ800; Japan) with forceps (World Precision Instruments, USA). After bone removal, the cochlea was transferred to a Transwell-Clear insert (six-well format; Corning, USA) with a permeable polyester membrane (0.4-μm pore size). The membranes were pre-coated with Celltak (Corning, USA) according to the manufacturer's protocol. The organ of Corti was then separated from stria vascularis and the modiolus and plated on the insert, with the hair cells facing up. Dissection medium was carefully removed, and 1.5 mL of otic culture medium (DMEM/F12 [Invitrogen, USA], 0.01% ampicillin [Sigma, USA], and 10% fetal bovine serum [Invitrogen, USA]) was added to the lower compartment under the insert membrane. On the following day, the medium on the insert was removed and they were transferred into an empty well. For the transduction, 200 μL of otic culture medium was added on the explant together with 70 μL of DMEM/F12 (Invitrogen, USA) containing 10^6, 5×10^6, or 10^7 particles of the stopGFP triple miRGE hairpin lentivector targeting p22phox. After 30 min of incubation at 37°C and 5% CO2, 1.5 mL of otic culture medium was added to the lower compartment. The medium was replaced with fresh otic culture medium on the following 2 days. Five days after the initial transduction, cochlear explants were either detached with trypsin for mRNA isolation or fixed for 10 min with 4% paraformaldehyde for immunostainings.

Immunostaining and Confocal Microscopy of Rat Organotypic Culture of Organ of Corti

Cochlear explants were fixed with 4% paraformaldehyde for 10 min at room temperature. Explants were transferred (by cutting the insert membrane) to a 24-well plate, washed three times with PBS, and permeabilized with 3% Triton X-100 for 30 min. Cochlear explants were immersed in a blocking buffer containing 2% BSA and 0.01% Triton X-100 for 1 h at room temperature. Explants were incubated with the anti-MyoVIIa (1:500, rabbit; Proteus, USA) antibody in blocking buffer overnight at 4°C. On the following day, tissues were rinsed three times with PBS and incubated with the secondary antibody anti-rabbit Alexa Fluor 488 (1:500; Invitrogen, USA) in blocking buffer for 2 h at room temperature. Explants were again washed three times with PBS and mounted on a glass slide with Fluoroshield containing DAPI (Sigma-Aldrich, USA). The labeled cells were visualized with a confocal laser-scanning microscope (Zeiss LSM710) equipped with a charge-coupled device (CCD) camera (Leica Microsystems) with a Planapochromat 10×/0.3 NA objective.

Knockdown of CCR5 in Humanized Mice Leukocytes

Human CD34 isolated from cord blood using magnetic beads (Miltenyi) were cultivated in activation medium (Cell Gro medium containing 20 ng/mL recombinant human [rh] stem cell factor (SCF), 20 ng/mL rh Flt3-L, 20 ng/mL rh interleukin-3 [IL-3], 20 ng/mL rh TPO1, 1% v/v penicillin-streptomycin [Penstrep]). The cells were seeded in a 24-well plate at 1.0×10^6 cells/mL for ~24 h at 37°C in activation medium for pre-stimulation. On the next day for transduction, Lentiblast B was added to the medium in a dilution of 1:1,000. Used MOI for transduction was 50. One well with 0.1×10^6 cells was not transduced and served as negative control. CD34 cells were cultivated for 48 h and then harvested except the transduction controls. The cells designated for transplantation were frozen and stored until transplantation of newborn NGS mice in liquid N_2. Newborn NGS mice were then irradiated with 1 Gy and then transplanted with 260,000 $CD34^+$ cells. Week 23 after birth, engraftment check was done by analyzing peripheral blood from the mice. CCR5 expression was then investigated at 28 weeks old using the following antibodies: huCD45 FITC (304006), CD3 AF700 (300424), CD4 PE-CY7 (300512), CD8 BV421 (301036), and CCR5 APC (359122) (or isocontrol #400611) from BioLegend.

Prediction of the MFE of Spacer Sequences

The MFE of spacers was calculated using RNA fold web server (Institute for Theoretical Chemistry, University of Vienna; http://rna.tbi.univie.ac.at/cgi-bin/RNAWebSuite/RNAfold.cgi). To allow comparison of MFE between spacers, obtained values were divided by the length of the spacer.

Calculation of the Efficiency of Concatenation of Triple Hairpin Concatenates

The concatenation efficiency of triple hairpin constructs (E) was calculated according to the formula $E = \ln(CP)/\ln(KP)$, where KP and CP are, respectively, the knockdown of CCR5 obtained with single and triple hairpin constructs. If $E = 3$, a fully additive effect of the hairpins is observed in the concatenate. If $E = 1$, the triple hairpin construct is as efficient as the single hairpin construct, and no additive effect of the hairpins concatenation is observed.

Statistical Analysis

Statistical analyses were performed using GraphPad Prism 5.04 (GraphPad Software, La Jolla, CA, USA). We used one-way ANOVA followed by Bonferroni's multiple comparison tests, as well as t tests (non-parametric, Mann-Whitney U test).

SUPPLEMENTAL INFORMATION

Supplemental Information includes five figures and six tables and can be found with this article online at https://doi.org/10.1016/j.omtn.2018.12.004.

AUTHOR CONTRIBUTIONS

F.R., K.H.K., and P.S. designed and analyzed the experiments and wrote the manuscript. F.R., O.C., M.C., M.P., M.R., and S.B. performed the experiments. R.M., M.A., R.F.S., and P.S. helped with the discussion of experiments and with writing of the manuscript.

CONFLICTS OF INTEREST

The authors declare no competing financial interests.

ACKNOWLEDGMENTS

The authors would like to thank Prof. Michael Pepper and all scientists involved in the Swiss South African Joint Research Program for helpful discussion. The authors also would like to thank Christina Müller, Audrey Fahrny, Maarja Grünbach, and Doris Russenberger

for technical assistance. This work was supported by funding from the Fondation Louis Jeantet "recherche translationnelle" (to K.H.K. and P.S.) and Swiss National Foundation grant 3200A0-103725 (to K.H.K.). R.F.S. and S.B. were supported by the clinical research focus program Human Hemato-Lymphatic Diseases of the University of Zurich.

REFERENCES

1. Winter, J., Jung, S., Keller, S., Gregory, R.I., and Diederichs, S. (2009). Many roads to maturity: microRNA biogenesis pathways and their regulation. Nat. Cell Biol. *11*, 228–234.
2. Sullenger, B.A., and Nair, S. (2016). From the RNA world to the clinic. Science *352*, 1417–1420.
3. Schwarz, D.S., Hutvágner, G., Du, T., Xu, Z., Aronin, N., and Zamore, P.D. (2003). Asymmetry in the assembly of the RNAi enzyme complex. Cell *115*, 199–208.
4. Chandradoss, S.D., Schirle, N.T., Szczepaniak, M., MacRae, I.J., and Joo, C. (2015). A dynamic search process underlies microRNA targeting. Cell *162*, 96–107.
5. Han, J., Lee, Y., Yeom, K.H., Nam, J.W., Heo, I., Rhee, J.K., Sohn, S.Y., Cho, Y., Zhang, B.T., and Kim, V.N. (2006). Molecular basis for the recognition of primary microRNAs by the Drosha-DGCR8 complex. Cell *125*, 887–901.
6. Chakraborty, C., Sharma, A.R., Sharma, G., Doss, C.G.P., and Lee, S.S. (2017). Therapeutic miRNA and siRNA: moving from bench to clinic as next generation medicine. Mol. Ther. Nucleic Acids *8*, 132–143.
7. Musacchio, T., and Torchilin, V.P. (2013). siRNA delivery: from basics to therapeutic applications. Front. Biosci. *18*, 58–79.
8. Boudreau, R.L., Martins, I., and Davidson, B.L. (2009). Artificial microRNAs as siRNA shuttles: improved safety as compared to shRNAs in vitro and in vivo. Mol. Ther. *17*, 169–175.
9. Grimm, D. (2011). The dose can make the poison: lessons learned from adverse in vivo toxicities caused by RNAi overexpression. Silence *2*, 8.
10. Maczuga, P., Lubelski, J., van Logtenstein, R., Borel, F., Blits, B., Fakkert, E., Costessi, A., Butler, D., van Deventer, S., Petry, H., et al. (2013). Embedding siRNA sequences targeting apolipoprotein B100 in shRNA and miRNA scaffolds results in differential processing and in vivo efficacy. Mol. Ther. *21*, 217–227.
11. Boudreau, R.L., Monteys, A.M., and Davidson, B.L. (2008). Minimizing variables among hairpin-based RNAi vectors reveals the potency of shRNAs. RNA *14*, 1834–1844.
12. Aiuti, A., Biasco, L., Scaramuzza, S., Ferrua, F., Cicalese, M.P., Baricordi, C., Dionisio, F., Calabria, A., Giannelli, S., Castiello, M.C., et al. (2013). Lentiviral hematopoietic stem cell gene therapy in patients with Wiskott-Aldrich syndrome. Science *341*, 1233151.
13. Biffi, A., Montini, E., Lorioli, L., Cesani, M., Fumagalli, F., Plati, T., Baldoli, C., Martino, S., Calabria, A., Canale, S., et al. (2013). Lentiviral hematopoietic stem cell gene therapy benefits metachromatic leukodystrophy. Science *341*, 1233158.
14. Myburgh, R., Cherpin, O., Schlaepfer, E., Rehrauer, H., Speck, R.F., Krause, K.H., and Salmon, P. (2014). Optimization of critical hairpin features allows miRNA-based gene knock-down upon single-copy transduction. Mol. Ther. Nucleic Acids *3*, e207.
15. Fowler, D.K., Williams, C., Gerritsen, A.T., and Washbourne, P. (2016). Improved knockdown from artificial microRNAs in an enhanced miR-155 backbone: a designer's guide to potent multi-target RNAi. Nucleic Acids Res. *44*, e48.
16. Lee, Y., Kim, M., Han, J., Yeom, K.H., Lee, S., Baek, S.H., and Kim, V.N. (2004). MicroRNA genes are transcribed by RNA polymerase II. EMBO J. *23*, 4051–4060.
17. Giry-Laterrière, M., Cherpin, O., Kim, Y.S., Jensen, J., and Salmon, P. (2011). Polyswitch lentivectors: "all-in-one" lentiviral vectors for drug-inducible gene expression, live selection, and recombination cloning. Hum. Gene Ther. *22*, 1255–1267.
18. Giry-Laterrière, M., Verhoeyen, E., and Salmon, P. (2011). Lentiviral vectors. Methods Mol. Biol. *737*, 183–209.
19. Liu, Y.P., and Berkhout, B. (2013). Design of lentivirally expressed siRNAs. Methods Mol. Biol. *942*, 233–257.
20. Stegmeier, F., Hu, G., Rickles, R.J., Hannon, G.J., and Elledge, S.J. (2005). A lentiviral microRNA-based system for single-copy polymerase II-regulated RNA interference in mammalian cells. Proc. Natl. Acad. Sci. USA *102*, 13212–13217.
21. Bourhill, T., Arbuthnot, P., and Ely, A. (2016). Successful disabling of the 5′ UTR of HCV using adeno-associated viral vectors to deliver modular multimeric primary microRNA mimics. J. Virol. Methods *235*, 26–33.
22. Yang, X., Marcucci, K., Anguela, X., and Couto, L.B. (2013). Preclinical evaluation of an anti-HCV miRNA cluster for treatment of HCV infection. Mol. Ther. *21*, 588–601.
23. Sun, D., Melegari, M., Sridhar, S., Rogler, C.E., and Zhu, L. (2006). Multi-miRNA hairpin method that improves gene knockdown efficiency and provides linked multi-gene knockdown. Biotechniques *41*, 59–63.
24. Seyhan, A.A. (2016). A multiplexed miRNA and transgene expression platform for simultaneous repression and expression of protein coding sequences. Mol. Biosyst. *12*, 295–312.
25. Liu, Y.P., Haasnoot, J., ter Brake, O., Berkhout, B., and Konstantinova, P. (2008). Inhibition of HIV-1 by multiple siRNAs expressed from a single microRNA polycistron. Nucleic Acids Res. *36*, 2811–2824.
26. Ruby, J.G., Jan, C.H., and Bartel, D.P. (2007). Intronic microRNA precursors that bypass Drosha processing. Nature *448*, 83–86.
27. Sibley, C.R., Seow, Y., Curtis, H., Weinberg, M.S., and Wood, M.J. (2012). Silencing of Parkinson's disease-associated genes with artificial mirtron mimics of miR-1224. Nucleic Acids Res. *40*, 9863–9875.
28. Osório, L., Gijsbers, R., Oliveras-Salvá, M., Michiels, A., Debyser, Z., Van den Haute, C., and Baekelandt, V. (2014). Viral vectors expressing a single microRNA-based short-hairpin RNA result in potent gene silencing in vitro and in vivo. J. Biotechnol. *169*, 71–81.
29. Hu, T., Chen, P., Fu, Q., Liu, Y., Ishaq, M., Li, J., Ma, L., and Guo, D. (2010). Comparative studies of various artificial microRNA expression vectors for RNAi in mammalian cells. Mol. Biotechnol. *46*, 34–40.
30. Kostic, C., Chiodini, F., Salmon, P., Wiznerowicz, M., Deglon, N., Hornfeld, D., Trono, D., Aebischer, P., Schorderet, D.F., Munier, F.L., and Arsenijevic, Y. (2003). Activity analysis of housekeeping promoters using self-inactivating lentiviral vector delivery into the mouse retina. Gene Ther. *10*, 818–821.
31. Rousset, F., Carnesecchi, S., Senn, P., and Krause, K.H. (2015). Nox3-targeted therapies for inner ear pathologies. Curr. Pharm. Des. *21*, 5977–5987.
32. Arimbasseri, A.G., Rijal, K., and Maraia, R.J. (2014). Comparative overview of RNA polymerase II and III transcription cycles, with focus on RNA polymerase III termination and reinitiation. Transcription *5*, e27639.
33. Salmon, P., Kindler, V., Ducrey, O., Chapuis, B., Zubler, R.H., and Trono, D. (2000). High-level transgene expression in human hematopoietic progenitors and differentiated blood lineages after transduction with improved lentiviral vectors. Blood *96*, 3392–3398.
34. Suen, T.C., and Goss, P.E. (2001). Identification of a novel transcriptional repressor element located in the first intron of the human BRCA1 gene. Oncogene *20*, 440–450.
35. Mechti, N., Piechaczyk, M., Blanchard, J.M., Jeanteur, P., and Lebleu, B. (1991). Sequence requirements for premature transcription arrest within the first intron of the mouse c-fos gene. Mol. Cell. Biol. *11*, 2832–2841.
36. Castellano, M., Golay, J., Mantovani, A., and Introna, M. (1992). Detection of a transcriptional block in the first intron of the human c-myb gene. Int. J. Clin. Lab. Res. *22*, 159–164.
37. Miura, H., Inoko, H., Tanaka, M., Nakaoka, H., Kimura, M., Gurumurthy, C.B., Sato, M., and Ohtsuka, M. (2015). Assessment of artificial miRNA architectures for higher knockdown efficiencies without the undesired effects in mice. PLoS ONE *10*, e0135919.
38. Mottet-Osman, G., Iseni, F., Pelet, T., Wiznerowicz, M., Garcin, D., and Roux, L. (2007). Suppression of the Sendai virus M protein through a novel short interfering RNA approach inhibits viral particle production but does not affect viral RNA synthesis. J. Virol. *81*, 2861–2868.
39. Jaquet, V., Marcoux, J., Forest, E., Leidal, K.G., McCormick, S., Westermaier, Y., Perozzo, R., Plastre, O., Fioraso-Cartier, L., Diebold, B., et al. (2011). NADPH oxidase (NOX) isoforms are inhibited by celastrol with a dual mode of action. Br. J. Pharmacol. *164*, 507–520.

OMTN, Volume *14*

Supplemental Information

Optimizing Synthetic miRNA Minigene Architecture for Efficient miRNA Hairpin Concatenation and Multi-target Gene Knockdown

Francis Rousset, Patrick Salmon, Simon Bredl, Ophélie Cherpin, Marta Coelho, Renier Myburgh, Marco Alessandrini, Michael Perny, Marta Roccio, Roberto F. Speck, Pascal Senn, and Karl Heinz Krause

Supplementary Material

Supplementary Fig. 1. Impact of the length and biophysical features of the spacer on the knockdown efficiency. Plots showing the correlation between CCR5 knockdown and (a) the minimum free energy/length or (b) the GC content of the spacers described in this figure.

Supplementary Fig. 2. Overall strategy for the design of efficient miRGE concatenate targeting NOX3 activity. (a) Candidate siRNA sequences are design of in silico and selected on their ability to knock-down the target mRNA (NOX3, $p22^{phox}$ or NOXO1). The most active siRNA sequence is then cloned in the miRGE backbone to construct the SMIG. Validation of the SMIG efficiency is verified on the same target expressing cell line. (b) Potency of 8 siRNA candidates, targeting mRNA encoding for the NOX3 complex (NOX3, $p22^{phox}$ and NoxO1) were assessed with respect to NOX3 activity by the amplex red method. (c) Effect of the most efficient sequence (targeting $p22^{phox}$) on NOX activity was verified by Western Blot. (d-e) Efficiency of the triple hairpin concatenate SMIG designed with the most active siRNA sequence was checked on the NOX3 expressing cell line either by QPCR (d) or by measurement of the production of ROS (e).

Supplementary Figure 3. Calculation of the efficiency of concatenation of the miRGE hairpins. (a) Table showing the formula allowing the calculation of the efficiency of concatenation (E) for GFP, MGST2 and H2B spacers. For the calculation of the concatenation efficiency, CCR5 knockdown with a single miRGE hairpin (knock-down potency (KP)) and with triple miRGE concatenate (concatenation potency (CP)) were considered. (b) Table summarizing the knock-down potency (KP), the concatenation efficiency (E) and their product, the general vector efficiency. (c) The former one is also displayed on the histogram.

Supplementary Figure 4. Engraftement rate of human hematopoietic stem cells in NOD/SCID mice, 23 weeks following transplantation. FACS plot showing human circulating leukocytes expressing human CD45 marker in (a) a mouse transplanted with untrannsduced HSC or (b) the 6 mice transplanted with HSC transduced with the triple hairpin concatenate targeting CCR5. Engraftment rate is between 12.4% and 44%.

Supplementary Figure 5. Identification of human CD4+ T cells from the transplanted mice blood. Total lymphocytes were selected according to their FSC/SSC coordinates (a). Following doublet elimination (b), human leukocytes were selected with human CD45 staining (c), then human lymphocytes with human CD3 antibody (d). Finally, CD4+ T cells were discriminated from CD8+ T cells with human CD4 and human CD8 antibodies (e).

Supplementary table 1. Biophysical features of the spacer sequences used in the vector constructs and putative function of translated proteins in the recipient cells.

Supplementary table 2. Lentivectors used in the study.

Supplementary table 3. Table of qPCR primers.

Supplementary table 4. Table of cloning primers.

Supplementary table 5. Table of DNA template for miRGE cloning.

Supplementary table 6. Sequences of the spacers

Supplementary Fig. 1

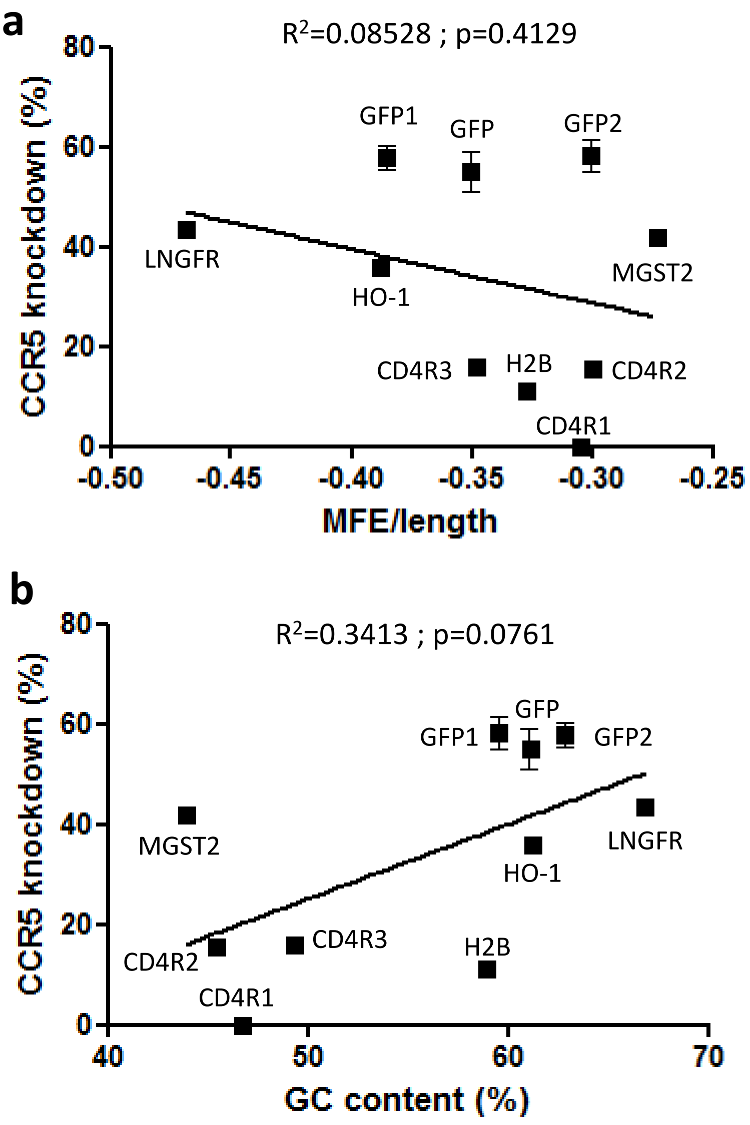

Supplementary Fig. 2

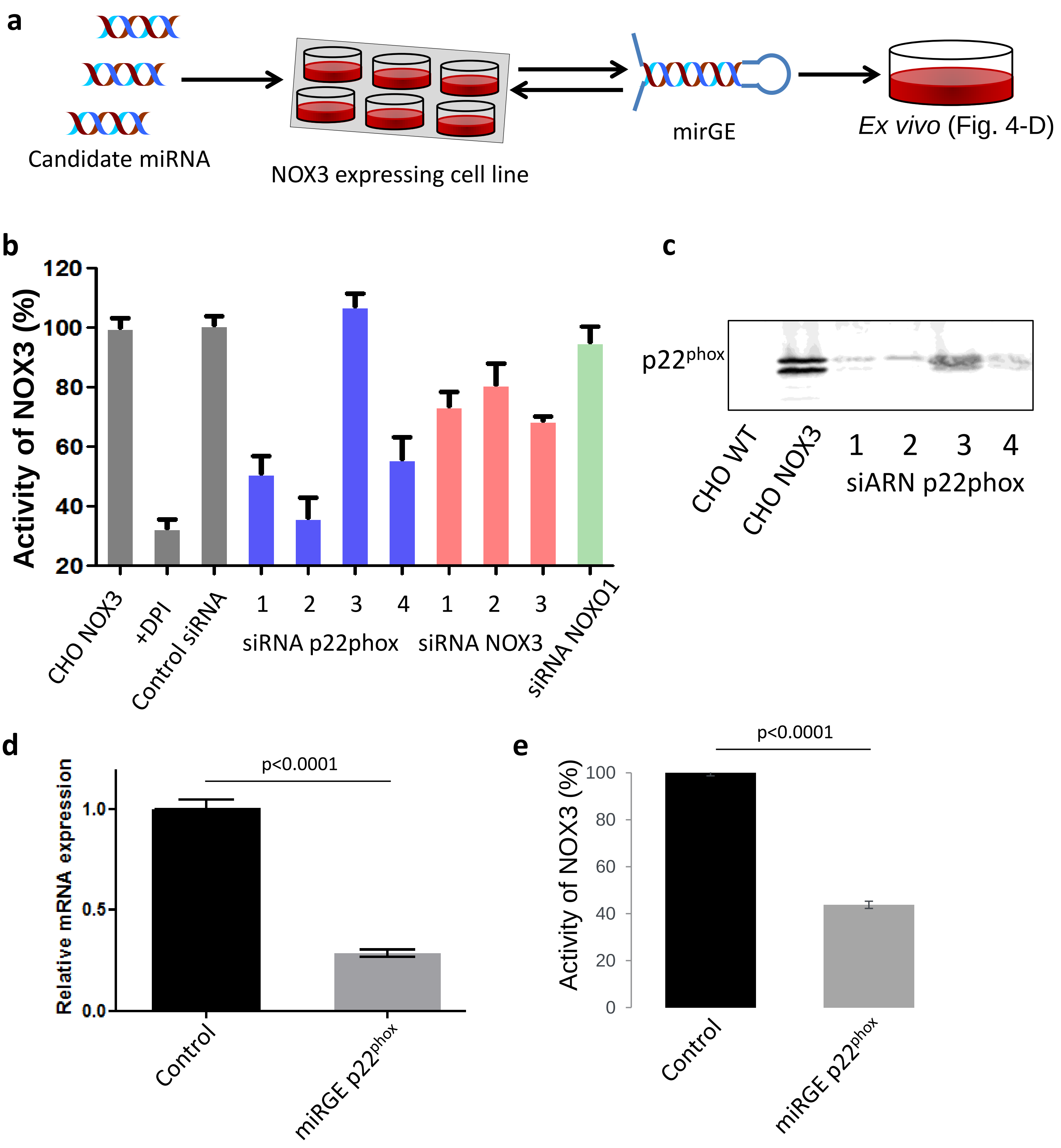

Supplementary Fig. 3

Concatenation potency = CP (%)<br>Knockdown potency = KP (%)	E= Concatenation efficiency with 3 hairpins
$CP=(KP)^{E}$<br>$E=\ln(CP)/\ln(KP)$	If E=3 concatenation efficiency is 100%<br>If E=1 concatenation efficiency is 0%

Supplementary Fig. 4

a

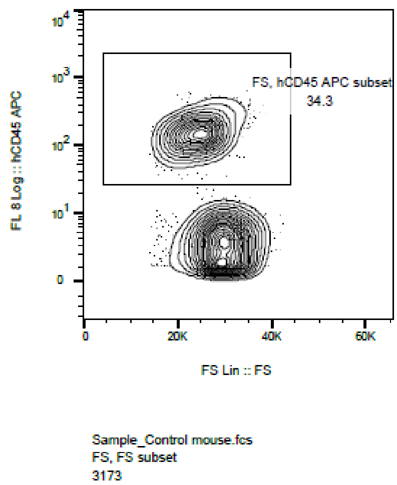

b

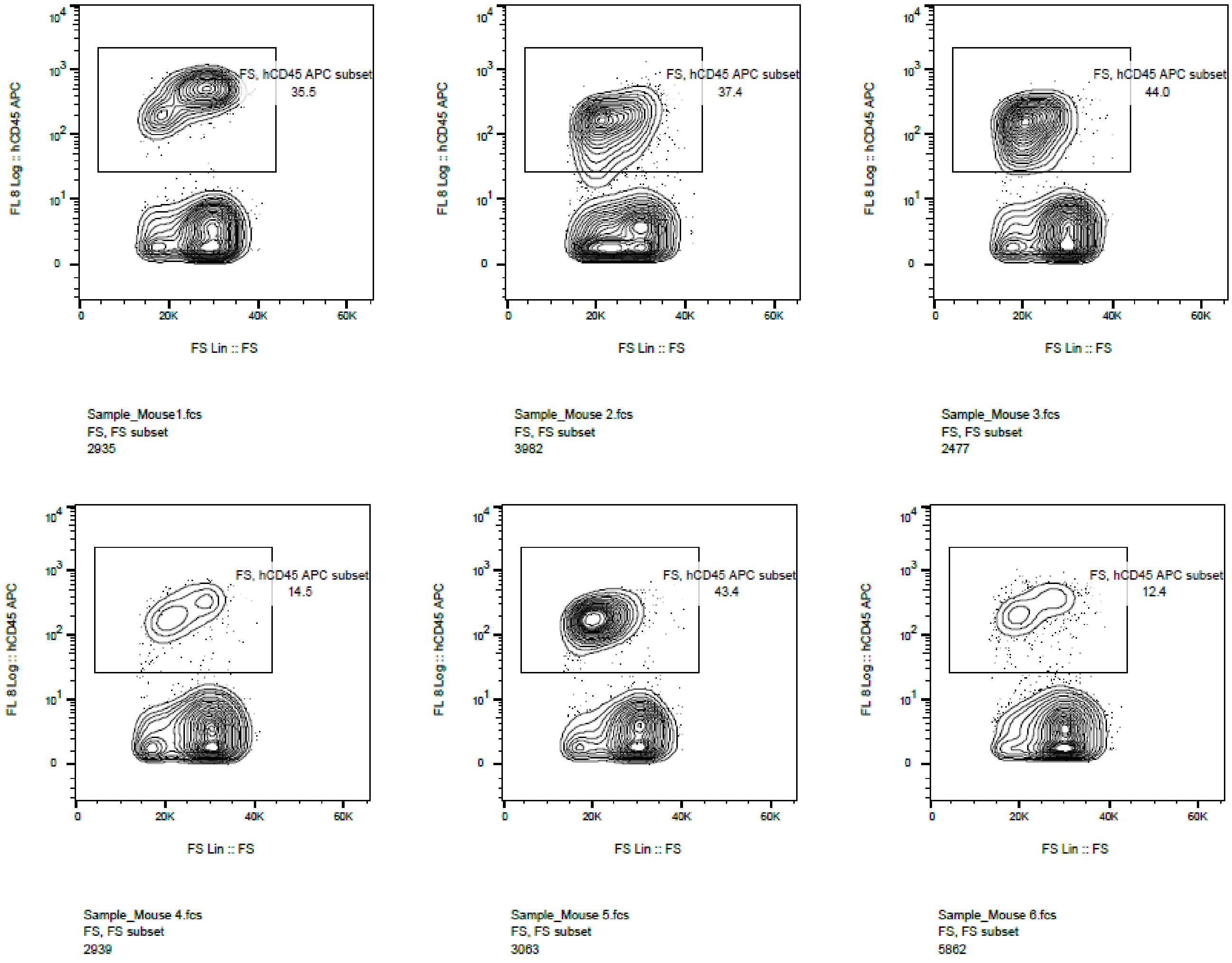

Supplementary Fig. 5

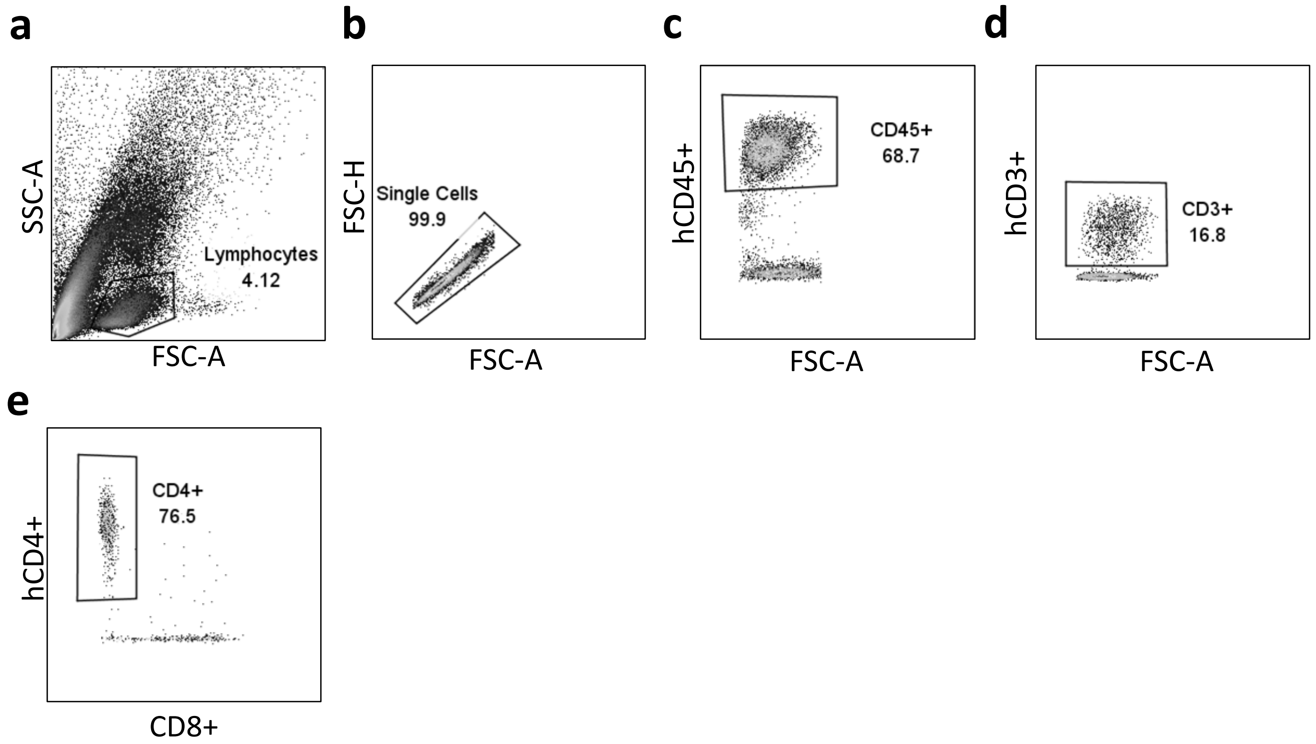

Supplementary table 1. Biophysical features of the spacer sequences

Spacer	Length (bp)	Predicted MFE Kcal/mol	% GC	coding	Comments
Green Fluorescent protein (GFP)<br>Truncated GFP1<br>GFP2	717<br>369<br>388	-264.6<br>-142.2<br>-116.4	61.11<br>62.87<br>59.53	YES	Reference spacer
Stop codons in the 3 reading frame after the start codon of GFP (stopGFP)	732	-	-	YES	non coding form
Microsomal Glutathion Transferase-2 (MGST2)	726	-197.7	43.92	YES	Resistance to Busulfan
Truncated form of the Nerve Growth Factor Receptor (deltaNGFR)	843	-397.7	66.86	YES	Pre-implantation selection
Heme Oxygenase (HO-1)	863	-336.2	61.24	YES	Cytoprotection
Histone2B (H2B)	378	-123.7	58.99	YES	Neutral
Amplicons from the 1st intron of the CD4 gene iCD41<br>iCD42<br>iCD43	793<br>620<br>380	-235.9<br>-189.2<br>-136	46.71<br>45.48<br>49.36	NO	Neutral ?

Supplementary table 2: Lentivectors used in this study

Lentivector	Titer (TU/mL)
pCWX-UBI-mcherry (control vector)	3.55E+06
pCWX-UBI-No spacer-mirGE CCR5-7-PGK-mCherry	1.02E+07
pCWX-UBI-GFP-mirGE CCR5-7-PGK-mCherry	4.66E+06
pCWX-UBI-mirGE CCR5-7-GFP-PGK-mCherry	1.17E+07
pCWX-UBI-MGST2-miRGE CCR5-7-PGK-mCherry	7.80E+05
pCWX-UBI-NGFR-miRGE CCR5-7-PGK-mCherry	1.94E+05
pCWX-UBI-CD4 R1-miRGE CCR5-7-PGK-mCherry	1.80E+06
pCWX-UBI-CD4 R2-miRGE CCR5-7-PGK-mCherry	2.76E+06
pCWX-UBI-CD4 R3-miRGE CCR5-7-PGK-mCherry	2.52E+06
pCWX-UBI-HO-1-miRGE CCR5-7-PGK-mCherry	1.60E+06
pCWX-UBI-H2B-miRGE CCR5-7-PGK-mCherry	1.22E+07
pCWX-UBI-stopGFP-miRGE CCR5-7-PGK-mCherry	3.54E+06
pCWX-UBI-GFPpart1-miRGE CCR5-7-PGK-mCherry	1.56E+06
pCWX-UBI-GFPpart2-miRGE CCR5-7-PGK-mCherry	1.59E+06
pCWX-UBI-MGST2-miRGE CYBA 222-PGK-mCherry	1.57E+06
pCWX-UBI-stopGFP-mirGE CYBA 222-PGK-mCherry	2.31E+06
pCWX-UBI- MGST2-mirGE-CCR5-777-PGK-mCherry	1.34E+07
pCWX-UBI- MGST2-mirGE-CCR5-777GFP-PGK-mCherry	1.20E+07
pCWX-UBI- MGST2-mirGE-CCR5-7777-PGK-mCherry	1.10E+05
pCWX-UBI- MGST2-mirGE-CCR5-7777GFP-PGK-mCherry	1.28E+07
pCWX-UBI-No spacer-mirGE CCR5-777-PGK-mCherry	6.81E+06
pCWX-UBI-mirGE CCR5-777-GFP-PGK-mCherry	1.10E+07
pCWX-UBI-H2B-miRGE CCR5-777-PGK-mCherry	5.06E+06
pCWX-UBI- stopGFP-mirGE-CCR5-777-PGK-mCherry	1.00E+06
pCWX-UBI- stopGFP-mirGE-CCR5-777GFP-PGK-mCherry	1.84E+05
pCWX-UBI- stopGFP-mirGE-CCR5-7777-PGK-mCherry	1.98E+06
pCWX-UBI- stopGFP-mirGE-CCR5-7777GFP-PGK-mCherry	1.12E+06
pCWX-UBI-stopGFP-miRGEGFP-PGK-mCherry	9.02E+05
pCWX-EFs-No spacer-mirGE CCR5-7-PGK-mCherry	9.25E+06
pCWX-EFs-GFP-mirGE CCR5-7-PGK-mCherry	1.21E+07
pCWX-EFs-GFP-mirGE CCR5-777-PGK-mCherry	1.27E+07
pCWX-EFs-mirGE CCR5-7-GFP-PGK-mCherry	6.82E+06
pCWX-EFs-mirGE CCR5-777-GFP-PGK-mCherry	4.75E+06
pCWX-EFs-No spacer-mirGE CCR5-777-PGK-mCherry	6.02E+06

Supplementary table 3: Table of qPCR primers used in this study

Amplicon	Primer forward 5’-3’	Primer reverse 5’-3’
Cyba (p22^{phox})	TGG ACG TTT CAC ACA GTG GT	TGG ACC CCT TTT TCC TCT TT
miRGE pri-miRNA	GGT GAT AGC AAT GTC AGC AGT GCC T	GTA GAG TAT GGT CAA CCT TAC TT
mature miRGE	LNA modified proprietary sequence (exiqon)	
GAG	GGA GCT AGA ACG ATT CGC AGT TA	GGT TGT AGC TGT CCC AGT ATT TGT C
EEF1a	TCC ACT TGG TCG CTT TGC T	CTT CTT GTC CAC AGC TTT GAT GA
GAPDH	TCC ATG ACA ACT TTG GCA TTG	CAG TCT TCT GGG TGG CAG TGA

Supplementary table 4: Table of cloning primers used in this study

Amplicon	Primer forward 5'-3' (AttB1-RcoRI)	Primer reverse 5'-3' (AttB2-XhoI)
GFP	GGG GAC AAG TTT GTA CAA AAA AGC AGG CTG AAT TCT GAG CAA GGG CGA GGA GCT GT	GGG GAC CAC TTT GTA CAA GAA AGC TGG GTC TCG AGC TTG TAC AGC TCG TCC ATG CCG
stopGFP	GGG GAC AAG TTT GTA CAA AAA AGC AGG CTT CTA GAA TGG ATG TAA GTA GGT GAG TGA GCA	GGG GAC CAC TTT GTA CAA GAA AGC TGG GTC TCG AGC TTG TAC AGC TCG TCC ATG CCG AGA
GFPpart1	GGG GAC AAG TTT GTA CAA AAA AGC AGG CTG AAT TCT GAG CAA GGG CGA GGA GCT GT	GGG GGC TCG AGT CGC CCT CGA ACT TCA CCT CG
GFPpart2	GGG GGG AAT TCC ACC CTG GTG AAC CGC ATC GA	GGG GAC CAC TTT GTA CAA GAA AGC TGG GTC TCG AGC TTG TAC AGC TCG TCC ATG CCG
lNGFR	GGG GAC CAC TTT GTA CAA GAA AGC TGG GTC TCG AGC TAG AGG ATC CCC CTG TTC CAC CT	GGG GAC AAG TTT GTA CAA AAA AGC AGG CTG AAT TCT CAC CAT GGG GGC AGG TGC CAC CGG
HO-1	GGG GAC AAG TTT GTA CAA AAA AGC AGG CTG AAT TCT CAC CAT GGA GCG TCC GCA ACC CGA	GGG GAC CAC TTT GTA CAA GAA AGC TGG GTC TAC AGC AAC TGT CGC CAC C
CD4R1	GGG GAC AAG TTT GTA CAA AAA AGC AGG CTG AAT TCT AAT AGT GAC CAC TCC TGG CTA ATT TTT GTA TTT TCA GTA GAG ATA GGG	GGG GAC CAC TTT GTA CAA GAA AGC TGG GTC TCG AGG GTG AAA CCC TTC TCT ACT AAA AAT ACA AAA TTA GCC GGG CAC A
CD4R2	-	GGG GAC CAC TTT GTA CAA GAA AGC TGG GTC TCG AGC CGC ACT CCA GCC TCG GCG ACA GAG CAA GAC TCT ATC TCA
CD4R3	-	GGG GAC CAC TTT GTA CAA GAA AGC TGG GTC TCG AGT CGG GAG TAC GAG ACC AGC CTG GCC AAC ATA GTG AAA TCC
H2B	GGG GAC AAG TTT GTA CAA AAA AGC AGG CTG AAT TCA TGC CAG AGC CAG CGA AGT C	GGG GAC CAC TTT GTA CAA GAA AGC TGG GTC TCG AGG TGT ACT TGG TGA CGG CCT TA
miRGE 1st hairpin	CAG AAG GGG ATC CAT CGA TAC TAG TGG TGA TAG CAA TGT CAG CAG TGC CT	AGT AGC TTC TAG AGT AGA GTA TGG TCA ACC TTA CTT
miRGE 2nd hairpin	CAG AAG GGG ATC CGG TGA TAG CAA TGT CAG CAG TGC CT	AGT AGC TAC TAG TGT AGA GTA TGG TCA ACC TTA CTT
miRGE 3rd hairpin	CAG AAG GCT CGA GGG TGA TAG CAA TGT CAG CAG TGC CT	AGT AGC TGG ATC CGT AGA GTA TGG TCA ACC TTA CTT

Supplementary table 5: Table of DNA template for miRGE cloning

	Template 5’-3’
miRGE CCR5-7	GGT GAT AGC AAT GTC AGC AGT GCC TTC ATA GAT TGG ACT TGA CAC TTG TGA AGC CAC AGA TGA AGT GTC AAG CCC AAT CTA TGC AAG TAA GGT TGA CCA TAC TCT AC
miRGE GFP	GGT GAT AGC AAT GTC AGC AGT GCC TAG TTC ACC TTG ATG CCG TTC TTG TGA AGC CAC AGA TGA AGA ACG GCA CCA AGG TGA ACC AAG TAA GGT TGA CCA TAC TCT AC
miRGE p22phox	GGT GAT AGC AAT GTC AGC AGT GCC TAC ATG GCC CAC TCG ATC TGC CCG TGA AGC CAC AGA TGG GGC AGA TCG CGT GGG CCA TGC AAG TAA GGT TGA CCA TAC TCT AC

Supplementary table 6: sequences of the spacers

stopGFP

aattcatggatgtaagtaggtgagtgagcaagggcgaggagctgttcaccggggtggtgcccatcctggtcgagctggacggcgacgtaaacggccacaagttcagcgtgtccggcgagggcgag ggcgatgccac
ctacggcaagctgaccctgaagttcatctgcaccaccggcaagctgcccgtgccctggcccaccctcgtgaccaccctgacctacggcgtgcagtgcttcagccgctaccccgaccacatgaagc agcacgacttc
ttcaagtccgccatgcccgaaggctacgtccaggagcgcaccatcttcttcaaggacgacggcaactacaagacccgcgccgaggtgaagttcgagggcgacaccctggtgaaccgcatcgagct gaagggcatcg
acttcaaggaggacggcaacatcctggggcacaagctggagtacaactacaacagccacaacgtctatatcatggccgacaagcagaagaacggcatcaaggtgaacttcaagatccgccacaac atcgaggacgg
cagcgtgcagctcgccgaccactaccagcagaacacccccatcggcgacggccccgtgctgctgcccgacaaccactacctgagcacccagtccgccctgagcaaagaccccaacgagaagcgcg atcacatggtc
ctgctggagttcgtgaccgccgccgggatcactctcggcatggacgagctgtacaagtaaagcggcctgaatcgccagtgtc

GFP1

aattcatggatgtaagtaggtgagtgagcaagggcgaggagctgttcaccggggtggtgcccatcctggtcgagctggacggcgacgtaaacggccacaagttcagcgtgtccggcgagggcgag ggcgatgccac
ctacggcaagctgaccctgaagttcatctgcaccaccggcaagctgcccgtgccctggcccaccctcgtgaccaccctgacctacggcgtgcagtgcttcagccgctaccccgaccacatgaagc agcacgacttc
ttcaagtccgccatgcccgaaggctacgtccaggagcgcaccatcttcttcaaggacgacggcaactacaagacccgcgccgaggtgaagttcgagggcgacc

GFP2

Aattccaccctggtgaaccgcatcgagctgaagggcatcgacttcaaggaggacggcaacatcctggggcacaagctggagtacaactacaacagccacaacgtctatatcatggccgacaagca gaagaacggca
tcaaggtgaacttcaagatccgccacaacatcgaggacggcagcgtgcagctcgccgaccactaccagcagaacacccccatcggcgacggccccgtgctgctgcccgacaaccactacctgagc acccagtccgc
cctgagcaaagaccccaacgagaagcgcgatcacatggtcctgctggagttcgtgaccgccgccgggatcactctcggcatggacgagctgtacaagtaaagcggcctgaatcgccagtgtc

H2B

Atgccagagccagcgaagtctgctcccgccccgaaaaagggctccaagaaggcggtgactaaggcgcagaagaaaggcggcaagaagcgcaagcgcagccgcaaggagagctattccatctatgt gtacaaggttc
tgaagcaggtccaccctgacaccggcatttcgtccaaggccatgggcatcatgaattcgtttgtgaacgacattttcgagcgcatcgcaggtgaggcttcccgcctggcgcattacaacaagcgc tcgaccatcac
ctccagggagatccagacggccgtgcgcctgctgctgcctggggagttggccaagcacgccgtgtccgagggtactaaggccgtcaccaagtacaccagcgctaag

NGFR

Aattctcaccatgggggcaggtgccaccggccgcgccatggacgggccgcgcctgctgctgttgctgcttctgggggtgtcccttggaggtgccaaggaggcatgccccacaggcctgtacacac acagcggtgag
tgctgcaaagcctgcaacctgggcgagggtgtggcccagccttgtggagccaaccagaccgtgtgtgagccctgcctggacagcgtgacgttctccgacgtggtgagcgcgaccgagccgtgcaa gccgtgcaccg
agtgcgtggggctccagagcatgtcggcgccgtgcgtggaggccgacgacgccgtgtgccgctgcgcctacggctactaccaggatgagacgactgggcgctgcgaggcgtgccgcgtgtgcgag gcgggctcggg
cctcgtgttctcctgccaggacaagcagaacaccgtgtgcgaggagtgccccgacggcacgtattccgacgaggccaaccacgtggacccgtgcctgccctgcaccgtgtgcgaggacaccgagc gccagctccgc
gagtgcacacgctgggccgacgccgagtgcgaggagatccctggccgttggattacacggtccacacccccagagggctcggacagcacagcccccagcacccaggagcctgaggcacctccaga acaagacctca
tagccagcacggtggcaggtgtggtgaccacagtgatgggcagctcccagcccgtggtgacccgaggcaccaccgacaacctcatccctgtctattgctccatcctggctgctgtggttgtgggc cttgtggccta
catagccttcaagaggtggaacaggggggatcctctagc

MGST2

aattcggcacgagggacttctgttccagagcaaaggtcattcagccgcttgaatcagccttttcccccccacccggtccccaactttgtttacccgataaggaaggtcagcattcaaagtcaagaa gcgccatttat
cttcccgtgcgctctacaaatagttccgtgagaaagatggccgggaactcgatcctgctggctgctgtctctattctctcggcctgtcagcaaagttatttgctttgcaagttggaaaggcaag attaaaataca
aagttacgcccccagcagtcactgggtcaccagagtttgagagagtatttcgggcacaacaaaactgtgtggagttttatcctatattcataattacattgtggatggctgggtggtatttcaac caagtttttgc
tacttgtctgggtctggtgtacatatatggccgtcacctatacttctggggatattcagaagctgctaaaaaacggatcaccggtttccgactgagtctggggattttggccttgttgaccctcc taggtgccctg
ggaattgcaaacagctttctggatgaatatctggacctcaatattgccaagaaactgaggcggcaattctaactttttctcttccctttaatgcttgcagaagctgttcccaccatgaaggtaat atggtatcatt
tgttaaataaaaataaagtctttattctgttaaaaaaaaaaaaaaaaaac

HO-1

Aattcatggagcgtccgcaacccgacagcatgccccaggatttgtcagaggccctgaaggaggccaccaaggaggtgcacacccaggcagagaatgctgagttcatgaggaactttcagaagggc caggtgacccg
agacggcttcaagctggtgatggcctccctgtaccacatctatgtggccctggaggaggagattgagcgcaacaaggagagcccagtcttcgcccctgtctacttcccagaagagctgcaccgca aggctgccctg
gagcaggacctggccttctggtacgggccccgctggcaggaggtcatcccctacacaccagccatgcagcgctatgtgaagcggctccacgaggtggggcgcacagagcccgagctgctggtggc ccacgcctaca
cccgctacctgggtgacctgtctgggggccaggtgctcaaaaagattgcccagaaagccctggacctgcccagctctggcgagggcctggccttcttcaccttccccaacattgccagtgccacc aagttcaagca
gctctaccgctcccgcatgaactccctggagatgactcccgcagtcaggcagagggtgatagaagaggccaagactgcgttcctgctcaacatccagctctttgaggagttgcaggagctgctga cccatgacacc
aaggaccagagcccctcacgggcaccagggcttcgccagcgggccagcaacaaagtgcaagattctgcccccgtggagactcccagagggaagcccccactcaacacccgctcccaggctccgct tctccgatggg
tccttacactcagctttctggtggcgacagttgctgtagggctttatgccatgtgac

iCD41

aattctaatagtgaccactcctggctaatttttgtattttcagtagagatagggtttcactatgttggccaggctggtctccaactcctgacctaaagtgatccacccaccttggtttcccaaag tgctgggatta
caggcgtgagccaccgtgcctggacatatatctatctttttttttttttgagatggagtctcgctctgttgcccaggctggagtgcagtggcgtgatttcggctcactgcaacctccgcctcccgg gttcaagtgat
tctcctgcctcagcctcccaagtagctgagattacagacgtgcgtcaccatgcccagctaatttttgtatttttagtagagatgggatttcactatgttggccaggctggtctcgtactcccgac ctcaggtgatc
cacttgccttggcctcccaaagtgctggaattacaggtgtgagccactgcatccggccttatatatctatcttgtctgtctgactgtctaatctaattcatctattttatctgtttatcttatct atcatctattt
atctaatctatctgtctgtatgtctgttttttttttgttttttttttttttttgagatagagtcttgctctgtcgccgaggctggagtgcggtggcgcgatctcagctcactgctgaacctccgc ctcctgggttc
taagcgattctcctgcctcaatctttggagtagctgggattacaggcccgtaccactgtgcccggctaattttgtatttttagtagagaagggtttcaccc

iCD42

aattctaatagtgaccactcctggctaatttttgtattttcagtagagatagggtttcactatgttggccaggctggtctccaactcctgacctaaagtgatccacccaccttggtttcccaaagtgctgggatta
caggcgtgagccaccgtgcctggacatatatctatctttttttttttttgagatggagtctcgctctgttgcccaggctggagtgcagtggcgtgatttcggctcactgcaacctccgcctcccgg gttcaagtgat
tctcctgcctcagcctcccaagtagctgagattacagacgtgcgtcaccatgcccagctaatttttgtatttttagtagagatgggatttcactatgttggccaggctggtctcgtactcccgac ctcaggtgatc
cacttgccttggcctcccaaagtgctggaattacaggtgtgagccactgcatccggccttatatatctatcttgtctgtctgactgtctaatctaattcatctattttatctgtttatcttatct atcatctattt
atctaatctatctgtctgtatgtctgttttttttttgttttttttttttttttgagatagagtcttgctctgtcgccgaggctggagtgcggc

iCD43

aattctaatagtgaccactcctggctaatttttgtattttcagtagagatagggtttcactatgttggccaggctggtctccaactcctgacctaaagtgatccacccaccttggtttcccaaag tgctgggatta
caggcgtgagccaccgtgcctggacatatatctatctttttttttttttgagatggagtctcgctctgttgcccaggctggagtgcagtggcgtgatttcggctcactgcaacctccgcctcccgg gttcaagtgat
tctcctgcctcagcctcccaagtagctgagattacagacgtgcgtcaccatgcccagctaatttttgtatttttagtagagatgggatttcactatgttggccaggctggtctcgtactcccgac
